# LncRNA CEBPA-DT promotes liver cancer metastasis through DDR2/β-catenin activation via interacting with hnRNPC

**DOI:** 10.1186/s13046-022-02544-6

**Published:** 2022-12-06

**Authors:** Yunshi Cai, Tao Lyu, Hui Li, Chang Liu, Kunlin Xie, Lin Xu, Wei Li, Hu Liu, Jiang Zhu, Yinghao Lyu, Xuping Feng, Tian Lan, Jiayin Yang, Hong Wu

**Affiliations:** 1 Liver Transplantation Center, State Key Laboratory of Biotherapy and Cancer Center, West China Hospital, Sichuan University and Collaborative Innovation Center of Biotherapy, Chengdu, 610041 China; 2 Laboratory of Liver Surgery, West China Hospital, Sichuan University, Chengdu, 610041 China; 3 Department of Hepatobiliary Pancreatic Tumor Center, Chongqing Key Laboratory of Translational Research for Cancer Metastasis and Individualized Treatment, Chongqing University Cancer Hospital, Chongqing, 400030 China; 4 Department of Plastic and Burns Surgery, West China Hospital, Sichuan University, Chengdu, 610041 China

**Keywords:** Hepatocellular carcinoma, CEBPA-DT, hnRNPC, DDR2, β-catenin, EMT

## Abstract

**Background:**

Hepatocellular carcinoma (HCC) is the world’s third leading cause of cancer-related death; due to the fast growth and high prevalence of tumor recurrence, the prognosis of HCC patients remains dismal. Long non-coding RNA CEBPA-DT, a divergent transcript of the CCAAT Enhancer Binding Protein Alpha (CEBPA) gene, has been shown to participate in multiple tumor progression. However, no research has established its cancer-promoting mechanism in HCC yet.

**Methods:**

CEBPA-DT was identified in human HCC tissues through RNA sequencing. The expression level of CEBPA-DT was assessed by quantitative real-time PCR. The biological effects of CEBPA-DT were evaluated *in vitro* and *in vivo* through gain or loss of function experiments. RNA fluorescence in situ hybridization (FISH), RNA immunoprecipitation (RIP) and RNA pull-down assays were applied to investigate the downstream target of CEBPA-DT. Immunofluorescence, subcellular protein fractionation, western blot, and co-immunoprecipitation were performed to analyze the subcellular location of β-catenin and its interaction with Discoidin domain-containing receptor 2 (DDR2).

**Results:**

CEBPA-DT was upregulated in human HCC tissues with postoperative distant metastasis and intimately related to the worse prognosis of HCC patients. Silencing of CEBPA-DT inhibited the growth, migration and invasion of hepatoma cells *in vitro* and *in vivo*, while enhancement of CEBPA-DT played a contrasting role. Mechanistic investigations demonstrated that CEBPA-DT could bind to heterogeneous nuclear ribonucleoprotein C (hnRNPC), which facilitated cytoplasmic translocation of hnRNPC, enhanced the interaction between hnRNPC and DDR2 mRNA, subsequently promoted the expression of DDR2. Meanwhile, CEBPA-DT induced epithelial-mesenchymal transition (EMT) process through upregulation of Snail1 via facilitating nuclear translocation of β-catenin. Using DDR2 inhibitor, we revealed that the CEBPA-DT induced the interaction between DDR2 and β-catenin, thus promoting the nuclear translocation of β-catenin to activate transcription of Snail1, contributing to EMT and HCC metastasis.

**Conclusions:**

Our results suggested that CEBPA-DT promoted HCC metastasis through DDR2/β-catenin mediated activation of Snail1 via interaction with hnRNPC, indicating that the CEBPA-DT-hnRNPC-DDR2/β-catenin axis may be used as a potential therapeutic target for HCC treatment.

**Supplementary Information:**

The online version contains supplementary material available at 10.1186/s13046-022-02544-6.

## Background

Primary liver cancer (PLC) is the sixth most common human cancer type and ranks third in cancer mortality worldwide [1]. Hepatocellular carcinoma (HCC), the predominant histological type of liver cancer, accounts for approximately 90% of the PLC [2]. Despite continuous improvement in detection and treatment, the long-term outcomes of HCC patients remain poor due to rapid progression and high prevalence of postoperative tumor recurrence and metastasis, with a five-year overall survival rate of less than 30% [3, 4]. Therefore, it is essential to illuminate the molecular mechanisms of HCC growth and progression to identify novel therapeutical targets.

Long non-coding RNAs (lncRNAs) are commonly referred to as a series of non-coding RNA transcripts longer than 200 nucleotides [5]. Recent studies have shown that lncRNAs are crucial for the initiation and development of many distinct cancer types, including breast cancer, lung cancer, and liver cancer [6–8]. LncRNAs can act as a sponge for messenger RNAs (mRNAs) and microRNAs (miRNAs) to perform regulatory roles. For instance, the lncRNA JPX activated Twist1 by competitively sponging miR-33a-5p and subsequently promoted EMT and lung cancer cell invasion [9]. The lncRNA TINCR regulated somatic tissue differentiation by interacting with a variety of differentiation mRNAs [10]. Although termed as "non-coding RNAs", recent studies have suggested additional roles for lncRNAs, including the capability of encoding peptides. For example, lnc-AP had been validated to encode the short peptide pep-AP that interacted with TALDO1 protein, thus inhibiting its expression, and sensitizing colorectal cancer cells to Oxaliplatin treatment [11]. Just as importantly, due to the comparatively large size that rendered lncRNAs with more complicated structures, a variety of lncRNAs exhibited the potential to interact with proteins and control the protein functions directly or indirectly. As an example, lncRNA HOTAIR acted as a scaffold for two different histone modification complexes, polycomb repressive complex 2 (PRC2) and LSD1/CoREST/REST complex, HOTAIR bound two different complexes allowed for RNA-mediated assembly of PRC2 and LSD1 as well as coordination of PRC2 and LSD1 targeting to chromatin for coupled H3 lysine 27 methylation and lysine 4 demethylation [12]. Although the functions and mechanisms of many lncRNAs have been uncovered, the roles of the majority of lncRNAs are still unknown.

The RNA binding protein heterogeneous nuclear ribonucleoprotein C (hnRNPC) is a member of the hnRNPs family [13]. A previous study illuminated that hnRNPC exerted its biological function by controlling the endogenous double-stranded RNA (dsRNA) splicing via binding with introns [14]. hnRNPC was predominantly located in the nucleus, it was able to translocate in the cytoplasm upon post-transcriptional stimulation [15]. Recent research has demonstrated that the interaction between lncRNA and hnRNPC could lead to the stabilization of mRNA [16]. Discoidin domain-containing receptor 2 (DDR2) represents a subclass of the receptor tyrosine kinase (RTKs) protein family, which is induced by collagen binding [17]. DDR2 has been validated to be relevant to multiple biological processes including osteoblast differentiation, fat metabolism and tumor microenvironment (TME) formation [18–20]. An early study showed that DDR1 gene contains a hnRNPA2 binding sequence [21]. However, the interaction between hnRNPC and DDR2 remains to be investigated.

In the present study, we identified lncRNA CEBPA-DT as an oncogene involved in HCC metastasis, which was closely associated with unfavorable outcomes. In addition, CEBPA-DT was found to interact with hnRNPC and facilitated its cytoplasmic translocation, thus enhancing the interaction between hnRNPC and DDR2 mRNA, subsequently promoting the expression of DDR2. Furthermore, CEBPA-DT induced EMT process through upregulation of Snail1 via facilitating nuclear export of β-catenin. Our study revealed a novel mechanism involved in lncRNA-regulated HCC metastasis.

## Materials and methods

### Cell lines

Hep3B, Huh7, SK-Hep1, PLC/PRF/5 and 293T cell lines were purchased from the National Collection of Authenticated Cell Cultures of the Chinese Academy of Science, Shanghai, China. SNU-499 cell line was obtained from ATCC (Manassas, VA, USA). All cell lines were validated through short tandem repeat (STR) analyses supplied by third-party biology services to ensure the authenticity of origin (Feiouer Biology Co., Ltd., Chengdu, China). Hep3B cells were cultivated in Minimum Essential Medium (HyClone, Logan, UT, USA) containing 10% FBS (Gibco), 1% penicillin/streptomycin (HyClone) at 37 °C and 5% CO2. The other cells were cultured in Dulbecco’s modified Eagle’s medium (HyClone, Logan, UT, USA) containing 10% FBS (Gibco), 1% penicillin/streptomycin under 37 °C and 5% CO2. Actinomycin D was obtained from Sigma-Aldrich. DDR2 inhibitor DDR2-IN-1 was purchased from MedChemExpress.

### Clinical samples

A total of 164 samples were obtained from identified HCC patients following curative hepatectomy (without preoperative treatment) at West China Hospital (WCH, Chengdu, China), among which 54 HCC tissues were with corresponding adjacent normal tissues. The patients were divided into two cohorts. Cohort 1 included 22 patients (11 patients with postoperative extrahepatic metastasis and 11 without extrahepatic metastasis). 22 HCC tumor tissues of cohort 1 were used to conduct RNA-seq and quantitative PCR validation. Samples of cohort 2 including 142 patients used for quantification of CEBPA-DT and analysis of the correlation between the expression of CEBPA-DT and the prognosis of surgically treated HCC patients. The protocols employed in this study were approved by the Ethical Review Committees of West China Hospital, and all patients provided written informed consent.

### RNA sequencing

For lncRNA sequencing, total RNA extracts of HCC tissues with or without extrahepatic metastasis (*n* = 11) were applied for RNA-seq to illuminate the differentially expressed lncRNAs. For the down-stream mRNA-analysis dataset, total RNA was extracted from siNC cells and siCEBPA-DT cells (*n* = 3). The detailed processes were previously described [22]. The differential expression analyses were performed with the edgeR package in R (v4.0.5) (www.r-project.org). The Benjamini–Hochberg method was used to alter the *p* values in order to control the False Discovery Rate (FDR). log2[fold change (FC)] > 1 and FDR < 0.05 were used as cutoff values for differentially expressed RNAs.

### The Cancer Genome Atlas (TCGA) data analysis

RNA-sequencing data and clinical information of 317 LIHC (Liver Hepatocellular Carcinoma) samples and 50 adjacent normal samples were obtained from cBioPortal (www.cbioportal.org). X-tile software (Yale University School of Medicine) was used to identify the optimal cutoff values for the expression levels of CEBPA-DT in WCH and TCGA datasets.

### RNA extraction and real-time quantitative PCR

Total RNA was extracted by using Trizol regent (Invitrogen, CA, USA) and Cell Total RNA Isolation Kit (Forgene, Chengdu, China) according to the manufacturer’s instructions. First-strand cDNA was generated using the HiScript II Reverse Transcriptase (Vazyme, Nanjing, China). Real-time quantitative PCR was performed with the ChamQ™ SYBR@ qPCR Master Mix (Vazyme, Nanjing, China). All reactions were performed in triplicate. U6 served as endogenous control. The relative expression was calculated using the comparative Ct (2^−ΔΔCT^) method. The primer sequences were listed in Table S5.

### Western blot analysis

Total protein was extracted by RIPA Lysis Buffer (Beyotime Biotechnology, Shanghai, China) and protease inhibitor cocktail (Thermo Fisher Scientific, CA, USA). The BCA Protein Assay Kit (Beyotime Biotechnology, Shanghai, China) was applied to detect protein concentration. Proteins were electrophoresed in sodium dodecyl sulfate–polyacrylamide gel electrophoresis (SDS-PAGE) and transferred onto PVDF membranes (Millipore, Massachusetts, USA). The membranes were incubated with primary antibodies and horseradish peroxidase (HRP)-conjugated secondary antibodies subsequently. The enhanced chemiluminescent (ECL) chromogenic substrate (4A Biotech, Beijing, China) was obtained for visualizing immunoreactivity. The intensity of signals was detected by ChemiDoc MP Imaging System (Bio-Rad, California, USA) and analyzed by the software of Image Lab 5.2 (Bio-Rad, California, USA). The primary antibodies used in this study were demonstrated in Table S4.

### Constructs and cell transfection

The full-length and truncated hnRNPC plasmids (Flag-hnRNPC) were constructed by GeneChem (Shanghai, China). The CTNNB1 plasmid was obtained from GeneCopoeia (Rockville, USA). The plasmids were transfected into 293 T cells using GenJet™ Plus reagent (SignaGen Laboratories, Maryland, USA). The specific small interfering RNA (siRNA).

oligonucleotides targeting hnRNPC, DDR2 and Snail1 were synthesized by GeneCodex (Wuhan, China). Genmute™ Reagent (SignaGen Laboratories, Maryland, USA) was used to perform transfection of siRNAs according to the manufacturer’s instructions. The sequences of siRNA were listed in Table S6.

### Construction of stable cell lines

The human CEBPA-DT sequence (NCBI Reference Sequence: NR_026887.2) was synthesized and cloned into lentiviral ORF expression vector pReceiver-Lv201 (GeneCopoeia, Rockville, USA), termed CEBPA-DT. Specific cDNA oligonucleotides targeting CEBPA-DT and DDR2 were cloned into the lentiviral shRNA expression vector psi-LVRU6GP (GeneCopoeia, Rockville, USA), named as shCEBPA-DT and shDDR2. All plasmids were transfected by using 293 T cells, viruses were produced in 293 T cells by using Lenti-Pac™ HIV packaging kit (GeneCopoeia, Rockville, USA) in accordance with the manufacturer’s instructions. The sequences of shRNA were listed in Table S6.

### RNA fluorescence in situ hybridization (FISH)

Cy3-labeled probes for detecting CEBPA-DT, 18S and U6 were synthesized by RiboBio (RiboBio Biotechnology, Guangzhou, China). RNA FISH was performed using the Fluorescent in situ Hybridization Kit (RiboBio Biotechnology) in accordance with the manufacturer’s instruction. Images were obtained with the AX10 imager A2 microscope (Carl Zeiss MicroImaging).

### Subcellular RNA fractionations

Nuclear and cytoplasmic RNA fractionations were performed using the PARIS™ Kit (Invitrogen, CA, USA) following the manufacturer’s instruction, analyzed by RT-qPCR successively, β-actin mRNA and U3 small nuclear RNA were used as the cytoplasmic and nuclear endogenous control, respectively.

### Subcellular protein fractionations

#### Nuclear and cytoplasmic protein fractionations were conducted by using the NE-PER™

Nuclear and Cytoplasmic Extraction Reagents (Thermo Fisher Scientific, CA, USA) in consistent with the protocols. Histon H3 and β-actin were used as the nuclear and cytoplasmic endogenous control, respectively.

### Tissue microarray and immunohistochemistry (IHC)

The tissue microarrays and IHC analyses were carried out as previously described [23]. Two independent pathologists examined the staining. IHC score was generated as follows: The staining intensity score (0, negative; 1, weak; 2, moderate; 3, strong) and the percentage score of positively stained cells (1 = less than 25% of the tumor cells; 2 = 25–50%; 3 = 50%-75%; 4 = more than 75%) were multiplied, ranging from 0–12. The antibodies for IHC were demonstrated in Table S4.

### Immunofluorescence (IF) analyses

4 × 10^4^ cells were plated on coverslips in 24-well plates. The cells were subsequently fixed with 4% paraformaldehyde at room temperature for 20 min, permeabilized with 0.2% Triton X-100 in PBS for 10 min, and then blocked with 5% BSA at room temperature for 1 h before being incubated with primary antibodies overnight at 4 °C. The cells were then rinsed with PBST (0.1% Tween-20 in PBS) before being treated with suitable fluorophore-conjugated secondary antibodies (1:1000, Thermo Fisher Scientific, CA, USA). After incubation with DAPI (Kaiji, Nanjing, China), the coverslips were mounted on slides and successively scanned with the AX10 imager A2 microscope (Carl Zeiss MicroImaging).

### *In vivo* cell behavior experiments

For CCK-8 experiments, 1000 cells/ 100μL culture medium were planted evenly in 96-well plates and incubated for four days. Cell viability was determined using the Cell Counting Kit-8 (CCK-8) (Beyotime Biotechnology, Shanghai, China) following the instructions. For the EdU cell proliferation assay, 2 × 10^4^ cells/ 500μL culture medium were seeded into the 24-well plate and incubated overnight. After being fixed with 4% paraformaldehyde at room temperature for 20 min, the EdU assay was performed using Cell-Light™ EdU *In Vitro* kit (RiboBio Biotechnology) in consistence with the protocols. Images were photographed using the OBSERVER D1/AX10 cam HRC microscope (Carl Zeiss MicroImaging) with a charge-coupled device (CCD) camera. For wound healing assays, 1 × 10^6^ cells per well were seeded into the 6-well plate. Confluent cell monolayers were injured with a 10μL plastic pipette tip. Image J (National Institutes of Health, USA) was used to calculate the proportion of wound-healing altered area after 24 or 48 h. Transwell chambers (Costar, Corning, NY) were placed in 24-well plates containing the culture medium with 10% FBS for migration and invasion experiments. Chambers were precoated with Matrigel for invasion experiments. The appropriate cell density for tests is 3 × 10^4^ cells per chamber for migration and 6 × 10^4^ cells per chamber for invasion. Image J was applied to count the number of cells in each chamber. According to the instructions, apoptosis experiments were carried out with Annexin V-FITC/PI or Annexin V-Alexa Fluor 647/PI apoptosis detection kit (4A biotech, Beijing, China). Cell cycle experiments were performed using the cell cycle detection kit (Keygen biotech, Nanjing, China) according to the protocols. Research Flow Cytometer (Beckman Coulter, CA, USA) was used for analyzing data of the cell apoptosis and cycle experiments.

### Animal xenograft experiments

The Animal Ethics Review Committees of West China Hospital approved the *in vivo* animal experiments. 6-week-old male BALB/c nude mice were provided by Charles River (Beijing, China) for *in vivo* studies. All mice were fed under pathogen-free conditions. All surgical procedures were conducted under anesthesia using sodium pentobarbital. 5 × 10^5^ hepatoma cells were injected subcutaneously into the right axilla of mice. Tumor size was measured every seven days by using a caliper and calculated with the formula of length × width^2^ × 0.52. The mice were sacrificed at 4 weeks following implantation before measurement of tumor weight. For liver orthotopic-implantation tumor models, 1 × 10^6^ hepatoma cells of the mice were injected into each liver of the mice. The mice were sacrificed 6 weeks after injection, the livers were photographed with the IVIS@ Lumina II system (Caliper Life Sciences, Hopkinton, MA). In addition, lung metastasis models were established by injecting 2 × 10^6^ hepatoma cells per mouse intravenously through the tail vein of the mice. After 6 weeks, the mice were sacrificed, and the lungs were photographed by the IVIS@ Lumina II system.

### RNA pull-down assays

ThE biotin-labeled CEBPA-DT was constructed *in vitro* with Ribo™ RNAmax-T7 Biotin-labeled Transcription Kit according to the manufacturer’s instructions (RiboBio Biotechnology, Guangzhou, China). The pull-down assays were then performed with Dynabeads™ Streptavidin Trial Kit (Thermo Fisher Scientific, CA, USA) in the protein lysates of HCCLM9 cells. The purified proteins were subsequently analyzed by mass spectrometry or western blot.

### Cross-Linked RNA immunoprecipitation (RIP) experiments

The hepatoma cells were pretreated with 0.3% formaldehyde to cross-link and then quenched with glycine solution. Cross-Linked RIP experiments were conducted with Magna RIP™ RNA binding Protein Immunoprecipitation Kit (Millipore, Massachusetts, USA) according to the manufacturer’s protocols. The precipitated RNAs were extracted and purified with phenol: chloroform: isoamyl alcohol (125: 24: 1). The purified RNAs were successively identified by RT-qPCR to evaluate the enrichment of target RNAs to the indicated proteins.

### Co-immunoprecipitation (Co-IP) assays

The Pierce Crosslink Magnetic IP/Co-IP Kit (Thermo Fisher Scientific, CA, USA) was utilized to conduct Co-IP experiments. In brief, the protein A/G magnetic beads were pre-bound to primary antibodies and incubated with cell lysates subsequently under 4℃ overnight. Then the antigens were dissociated from the antibody-beads complex with a low-pH elution buffer. The eluents were identified by western blot assays.

### Luciferase reporter experiments

For TOP/FOP Flash assay, the HCCLM9 cells were pre-seeded in a 24-well plate before transfection. The pRL-SV40-N plasmid (Beyotime Biotechnology, Shanghai, China) plus either the TOP-Flash or FOP-Flash plasmid (Beyotime Biotechnology, Shanghai, China) were co-transfected into the cells by using the Lipofectamine 3000 transfection reagent (Invitrogen, CA, USA). After 48 h, the luciferase activities were assessed with the Dual-Luciferase Reporter Assay Kit (Vazyme, Nanjing, China) in accordance with the instructions. The results were calculated by TOP/FOP Flash activities. For Snail1 promoter activity evaluation, the promoter region of Snail1 was cloned into firefly or renilla luciferase vector and then co-transfected into hepatoma cells with Lipofectamine 3000 transfection reagent. Detection of luciferase activities was performed after 48 h. The relative luciferase activities were demonstrated as firefly/renilla luciferase activities.

### Chromatin immunoprecipitation (ChIP) assays

Hepatoma cells were previously cross-linked with 1% paraformaldehyde and quenched with glycine solution subsequently. The ChIP assays were conducted according to Pierce Magnetic ChIP Kit (Thermo Fisher Scientific) instructions. Normal IgG (Millipore) and anti-β-catenin antibodies were utilized for immunoprecipitation. Purified DNAs were quantified by real-time PCR to evaluate the binding sites of the Snail1 promoter region. The sequences of primers were listed in Table S5.

### Statistical analysis

All the statistical analyses were conducted with EmpowerStats (www. empowerstats.net) and the software of GraphPad Prism 8. Data was shown as mean ± standard deviation (SD) or cases (percentage). The student’s t-test was used for the investigation of continuous variables; chi-square test was used for categorical variables. Kaplan–Meier method was utilized to plot the survival curves, and the log-rank test was used to test the differences. Pearson correlation analyses were applied to assess the correlations. Cox proportional hazards regression models were used to evaluate underlying prognostic factors to OS and RFS. Risk factors with *P* values less than 0.2 in univariate analyses were selected for the multivariate regression models. Statistically significance was considered as a two-tailed *P* value < 0.05.

## Results

### CEBPA-DT is upregulated in HCC tissues and correlated with patient prognosis

**Fig. 1:**
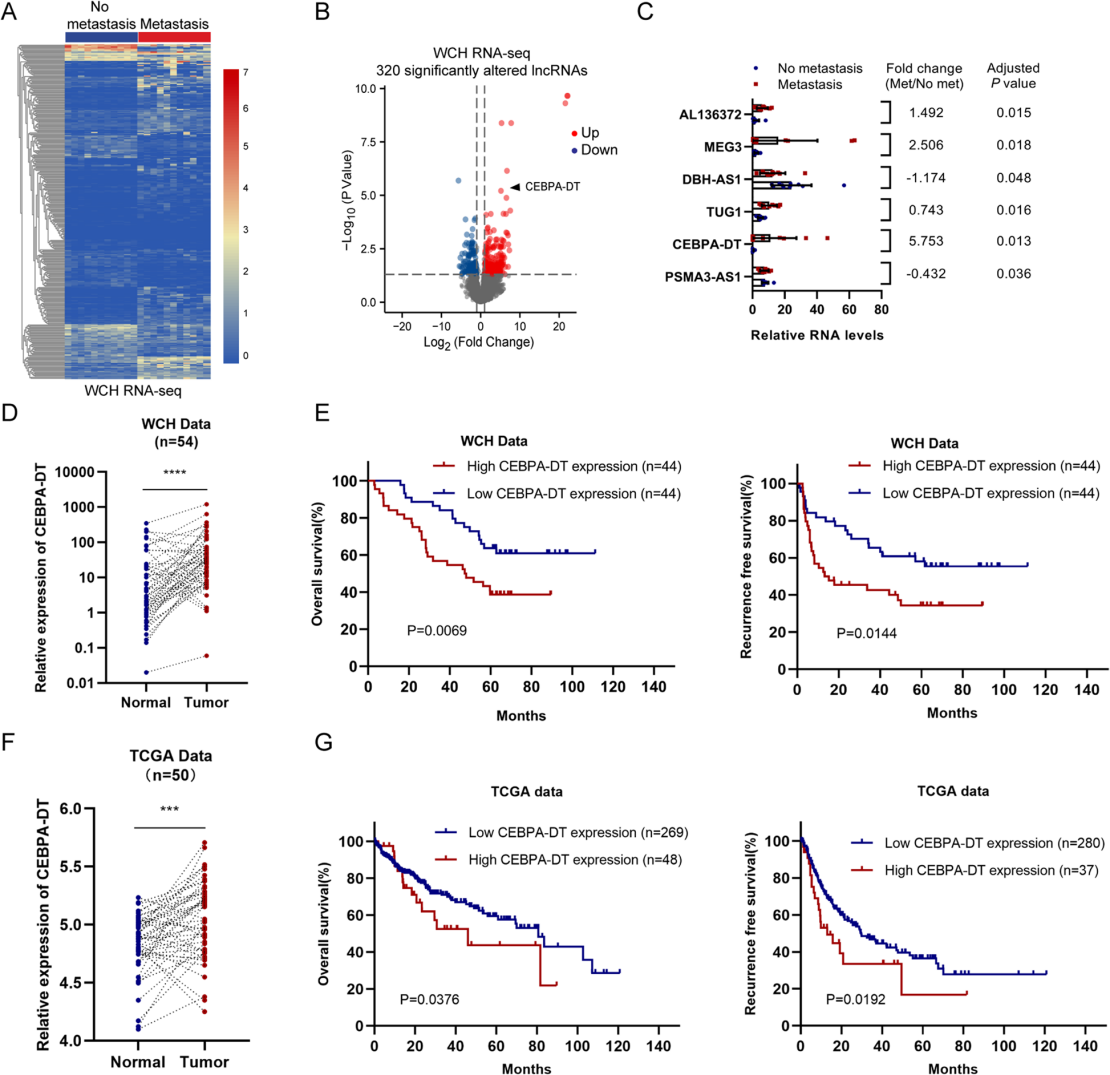
CEBPA-DT is upregulated in HCC with extrahepatic metastasis and associated with patient prognosis. **A** Clustering plot of differential lncRNA profiles between HCC tissues with or without extrahepatic metastasis. **B** Volcano plot of differential lncRNA profiles between HCC tissues with or without extrahepatic metastasis. **C** Relative RNA levels of 6 candidate lncRNAs in 22 HCC tissues with or without extrahepatic metastasis. **D** Relative CEBPA-DT expression levels in 54 paired HCC tissues and non-tumor tissues in West China Hospital cohort. **E** Kaplan–Meier curves showed relationship between CEBPA-DT expression and overall survival (left) or recurrence-free survival (right) in 88 HCC patients from West China Hospital cohort. **F** Relative CEBPA-DT expression levels in 50 paired HCC tissues and non-tumor tissues in TCGA cohort. **G** Kaplan–Meier curves showed relationship between CEBPA-DT expression and overall survival (left) or recurrence-free survival (right) in 317 HCC patients from TCGA LIHC cohort.  *p < 0.05, **p < 0.01, ***p < 0.001

In order to screen the lncRNAs that are associated with the metastasis of hepatocellular carcinoma, RNA-seq was performed to analyze the differentially expressed genes between 11 HCC tissues from patients with post-surgically extrahepatic metastasis and 11 HCC tissues obtained from patients without postoperative metastasis or recurrence, 320 significant altered lncRNAs were identified (Fig. 1A). 6 lncRNAs (CEBPA-DT, AL136372, MEG3, DBH-AS1, TUG1, PSMA3-AS1) with the highest abundance (average FPKM > 5) in the 11 HCC tissues from patients suffered from extrahepatic metastasis were identified. CEBPA-DT was selected as the candidate lncRNA for further study because it was the most significantly upregulated target (log_2_(Foldchange) = 5.753, *P* = 0.013) among the previously mentioned 6 candidate lncRNAs (Fig. 1B, C). Additionally, we examined the expression levels of CEBPA-DT in a cohort of 54 HCC tissues with the corresponding normal liver tissues. The RT-qPCR analyses showed that the expressions of CEBPA-DT in HCC tissues were significantly higher than that in the paired normal liver tissues (Fig. 1D). Similar results were likewise observed in the 50 paired HCC and normal tissues in TCGA LIHC data (Fig. 1F).

To further assess the prognostic value of the CEBPA-DT expression level in HCC patients, the expression levels of CEBPA-DT were measured in a cohort of 88 HCC tissues. Patients were classified into the high CEBPA-DT group (*n* = 44) and low CEBPA-DT group (*n* = 44) conforming to the expressions of CEBPA-DT in HCC tissues. The baseline characteristics of these 88 HCC patients were showed in Table S1. A higher CEBPA-DT expression level was significantly correlated to more elevated serum Alpha-fetoprotein (AFP) level, larger tumor size, poor tumor differentiation and microvascular invasion (MVI), suggesting that higher CEBPA-DT expression was associated with HCC growth and metastasis. Kaplan–Meier survival curves illustrated that the HCC patients in the high CEBPA-DT level group were significantly related to inferior overall survival (OS) and recurrence-free survival (RFS) than those patients with low levels of CEBPA-DT (Fig. 1E). Consistently, identical results were obtained from the TCGA LIHC cohort (Fig. 1G). In the univariate analysis, positive HBsAg status, poor tumor differentiation, tumor size > 5 cm, presence of MVI, AFP > 400 ng/μL, distant metastasis and high CEBPA-DT expression were identified as potential risk factors associated with dismal OS and RFS of HCC patients (Table S2). These variables were further included in the multivariate regression model. The results showed that the presence of MVI and high CEBPA-DT expression were independent risk factors for both OS and RFS (Table S3). Furthermore, in HCC patients with different clinical and pathological features, a high expression level of CEBPA-DT remained the risk factor for the OS as well as the RFS (Fig. S7A, B).

As presented in the National Center for Biotechnology Information (NCBI) database, there is only one annotated transcript (NR_026887.2, Figure S1A) for CEBPA-DT gene. Coding potential analyses were performed with the open reading frame finder (www.ncbi.nlm.nih.gov/orffinder; Figure S1B) and Coding-Potential Assessment Tool (CPAT) (http://lilab.research.bcm.edu/cpat/; Figure S1C) [24]. Both analyses indicated that the CEBPA-DT was with limited coding capacity. Next, subcellular RNA fractionation combined with RT-qPCR showed that CEBPA-DT was predominantly distributed in the nuclear of the hepatoma cell (Figure S2B).

Collectively, these findings suggested that CEBPA-DT is a highly abundant lncRNA that is significantly upregulated in HCC with extrahepatic metastasis. Furthermore, CEPBA-DT is a potential prognostic indicator for HCC patients.

### CEBPA-DT promotes proliferation and metastasis of hepatoma cells *in vitro* and *in vivo*

**Fig. 2:**
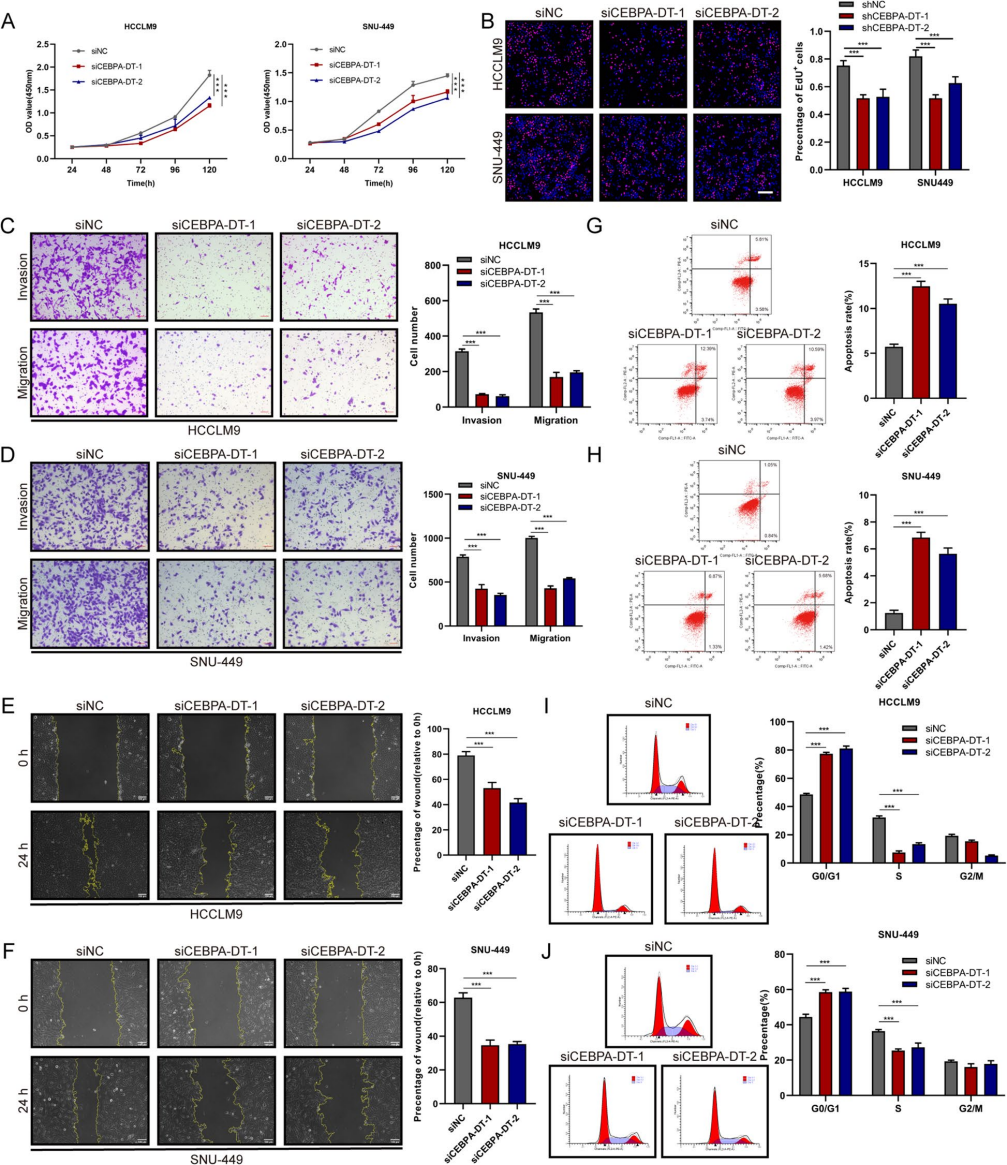
Silencing of CEBPA-DT represses tumor growth and metastasis of hepatoma cells *in vitro.* **A** Cell viability of indicated HCCLM9 and SNU-449 cells were measured by CCK-8 assays. **B** Cell proliferation of indicated HCCLM9 and SNU-449 cells were measured by EdU assays. Scale bar, 200 μm. **C-D** Cell migration and invasion capacity of indicated HCCLM9 and SNU-449 cells were measured by transwell migration and matrigel invasion assays. Scale bar, 100 μm. **E–F** Cell migration capacity of indicated HCCLM9 and SNU-449 cells were measured by scratch wound-healing assays. Scale bar, 100 μm. **G-H** Cell apoptosis rates in indicated HCCLM9 and SNU-449 cells were measured by flow cytometry. **I-J** Cell-cycle distribution of indicated HCCLM9 and SNU-449 cells were measured by propidium iodide staining followed by flow cytometry. Date are presented as mean ± SD; *n* = 3. Student’s t test was used. *p < 0.05, **p < 0.01, ***p < 0.001

**Fig. 3:**
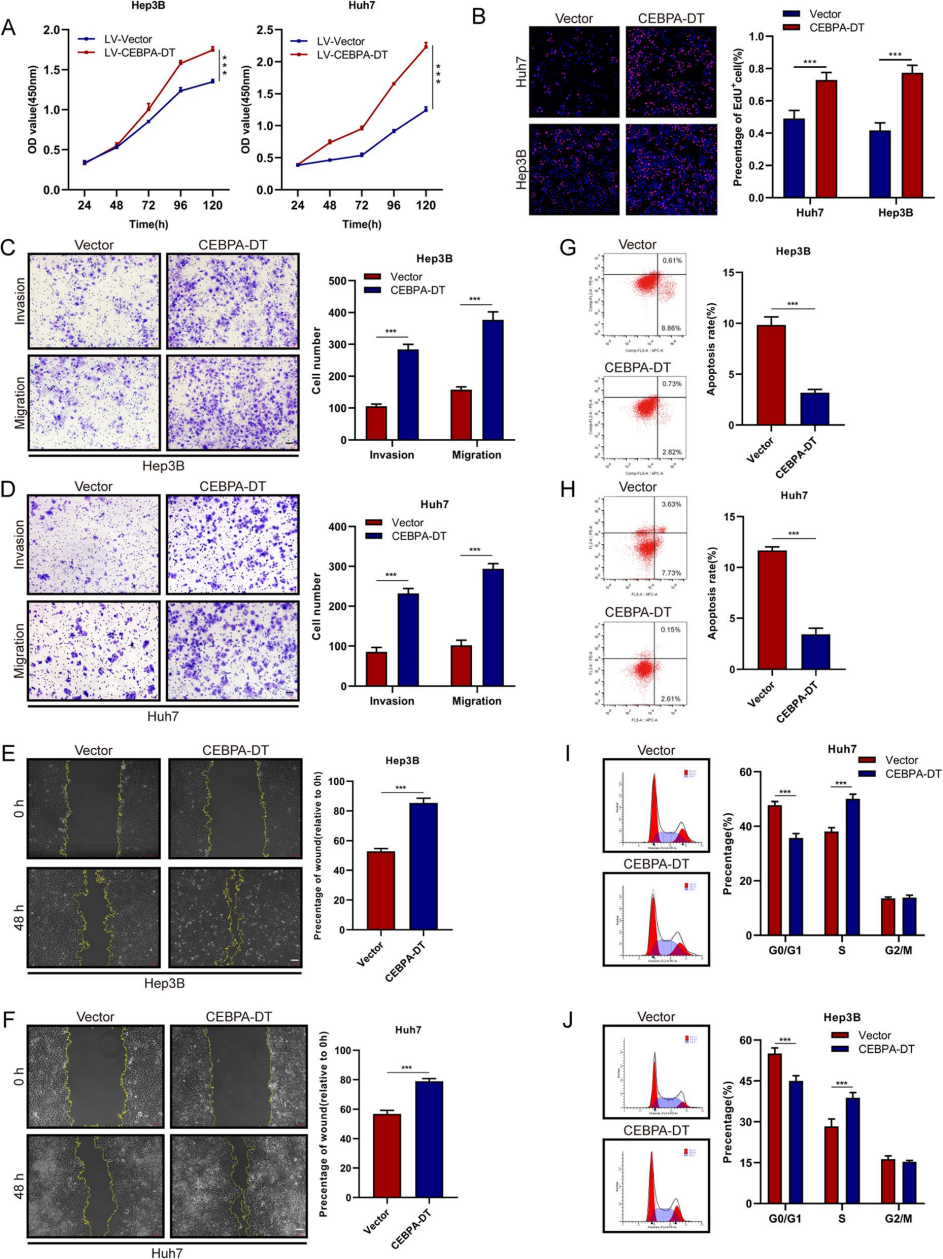
CEBPA-DT promotes tumor growth and metastasis of hepatoma cells *in vitro.* **A** Cell viability of indicated Hep3B and Huh7 cells were measured by CCK-8 assays. **B** Cell proliferation of indicated Hep3B and Huh7 cells were measured by EdU assays. Scale bar, 200 μm. **C-D** Cell migration and invasion capacity of indicated Hep3B and Huh7 cells were measured by transwell migration and matrigel invasion assays. Scale bar, 100 μm. **E–F** Cell migration capacity of indicated Hep3B and Huh7 cells were measured by scratch wound-healing assays. Scale bar, 100 μm. **G-H** Cell apoptosis rates in indicated Hep3B and Huh7 cells were measured by flow cytometry. **I-J** Cell-cycle distribution of indicated Hep3B and Huh7 cells were measured by propidium iodide staining followed by flow cytometry. Date are presented as mean ± SD; *n* = 3. Student’s t test was used. *p < 0.05, **p < 0.01, ***p < 0.001

Seven human hepatoma cell lines were selected to measure the expression levels of CEBPA-DT by RT-qPCR, the results exhibited that the CEBPA-DT expression levels were higher in HCCLM9, SNU-449 and SK-Hep1 than that in Huh7, Hep3B and PLC/PRF/5 (Figure S2A). To determine whether CEBPA-DT serves as an oncogenic lncRNA, small interfering RNAs (siRNAs) against the sequence of CEBPA-DT were used to knock down the CEBPA-DT expression in HCCLM9 and SNU-449 cells (Figure S2C). Moreover, we established stable CEBPA-DT knockdown cells (shCEBPA-DT-1) in HCCLM9 (Figure S2D). *In vitro* functional studies demonstrated that silencing CEBPA-DT significantly inhibited cell proliferation of HCCLM9 and SNU-449 cells (Fig. 2A, B). Meanwhile, the transwell migration, invasion and wound-healing migration assays showed that cell motility was dramatically suppressed after down-regulation of CEBPA-DT in hepatoma cells (Fig. 2C, D, E, F). In addition, Flow cytometry was applied to investigate the cell apoptosis and cell cycle distribution, it was shown that depletion of CEBPA-DT markedly increased the apoptosis rate of hepatoma cells (Fig. 2G, H). In the meantime, an increased percentage of hepatoma cells in the G0/G1 phase and decrease of that in the S phase were observed after silencing of CEBPA-DT (Fig. 2I, J). Next, stable hepatoma cell lines that overexpressed CEBPA-DT were constructed via the lentiviral infections in Huh7, Hep3B and SK-Hep1 cells (Figure S2E). Overexpression of CEBPA-DT significantly improved the growth capacity of Hep3B and Huh7 cells (Fig. 3 A, B). A marked enhancement of cell motility was observed in CEBPA-DT overexpressing hepatoma cells (Fig. 3C, D, E, F). The cell apoptosis assays exhibited that CEBPA-DT overexpression reduced the apoptosis rate of Huh7 and Hep3B cells (Fig. 3G, H). The cell cycle analyses revealed that CEBPA-DT overexpression increased the S fraction and decreased the G0/G1 fraction (Fig. 3I, J).

**Fig. 4:**
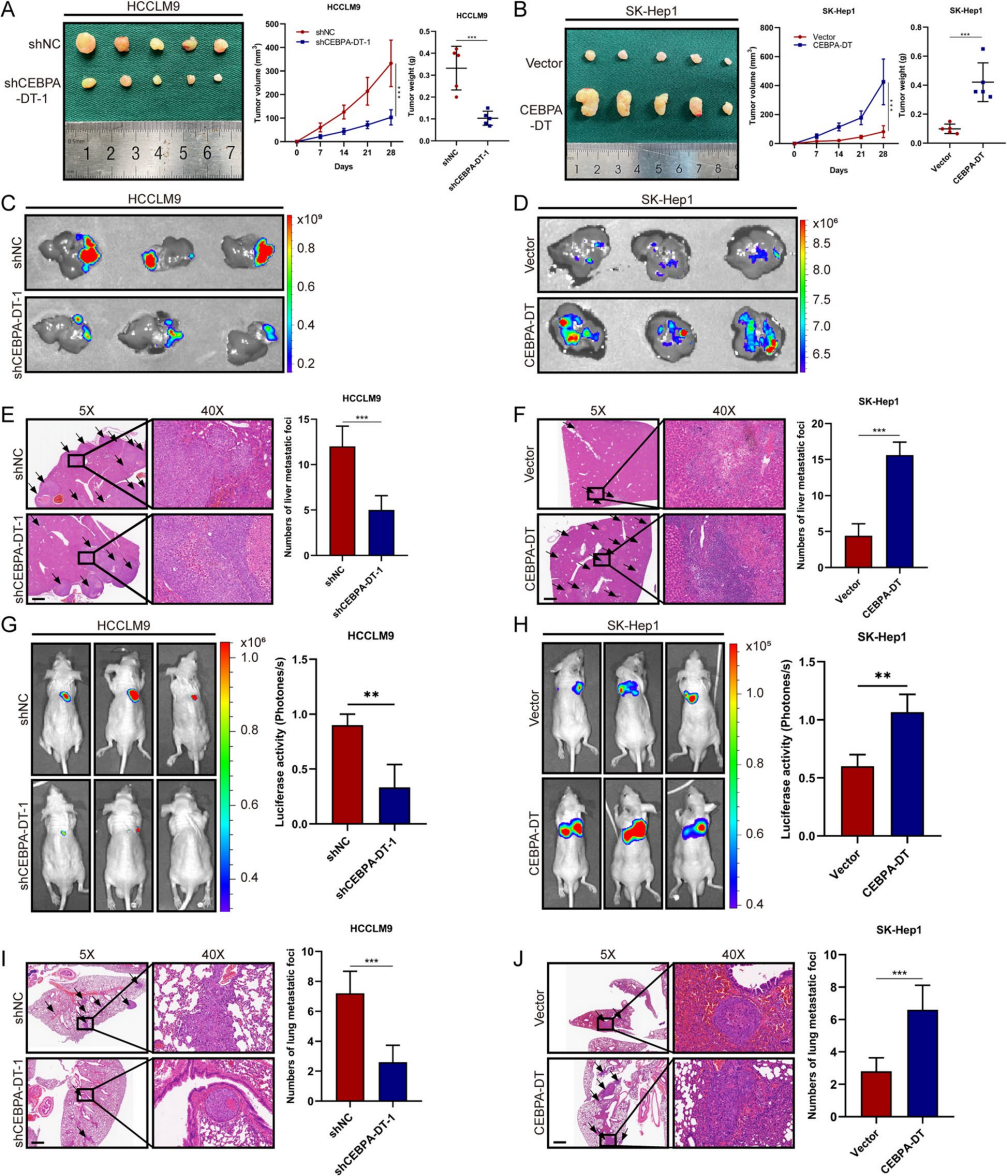
CEBPA-DT promotes tumor growth and metastasis *in vivo*. **A-B** Tumor volume and weight of subcutaneous xenografts in nude mice injected with indicated HCCLM9 and SK-Hep1 cells. **C-D** Representative images (fluorescence) of intrahepatic metastatic nodules in liver orthotopic-implantation tumor models with indicated HCCLM9 and SK-Hep1 cells. **E–F** Representative microscopic views of HE staining and the corresponding statistical analyses of intrahepatic metastatic nodules (black arrows) in liver orthotopic-implantation tumor models injected with indicated HCCLM9 and SK-Hep1 cells. Scale bar, 50 μm. **G-H** Representative bioluminescent images and the corresponding statistical analyses of bioluminescent tracking plots for lungs of nude mice injected with indicated HCCLM9 and SK-Hep1 cells through tail vein. **I-J** Representative microscopic views of HE staining and the corresponding statistical analyses of lung metastatic nodules (black arrows) in nude mice injected with indicated HCCLM9 and SK-Hep1 cells through tail vein. Scale bar, 50 μm. Date are presented as mean ± SD; *n* = 3. Student’s t test was used. *p < 0.05, **p < 0.01, ***p < 0.001

We further determined the effect of CEBPA-DT on tumorigenicity *in vivo.* CEBPA-DT silencing HCCLM9 cells and CEBPA-DT overexpressing SK-Hep1 cells were established and injected subcutaneously into nude mice. A remarkable decrease in tumor volume and weight were observed when CEBPA-DT was knocked down, and a contrary result was obtained when CEBPA-DT was overexpressed (Fig. 4A, B), suggesting that CEBPA-DT promoted tumor growth *in vivo*. To further evaluate the effect of CEBPA-DT on tumor metastasis *in vivo*, liver orthotopic-implantation models and lung metastasis models were established. After six weeks, lower fluorescence intensities of GFP were observed in CEBPA-DT silencing group than that in the control group both in the liver (Fig. 4C) and lung (Figure S2F), opposite results were exhibited when CEBPA-DT was overexpressed (Fig. 4D, Figure S2G). The IVIS photographing results demonstrated that CEBPA-DT knockdown group was associated with weaker bioluminescence signal intensities in the lung than the control group (Fig. 4G); meanwhile, CEBPA-DT overexpression enhanced the bioluminescence activities in lung compared to the empty vector group (Fig. 4H). The results of hematoxylin and eosin (HE) staining exhibited that the number of metastatic foci of CEBPA-DT silencing group was decreased both in the liver (Fig. 4E) and lung (Fig. 4I). In contrast, increased numbers of liver and lung metastatic foci were observed in CEBPA-DT overexpression group compared to the control group (Fig. 4F, J). To sum up, these observations indicated that CEBPA-DT was a cancer-promoting lncRNA, which facilitated the proliferation and metastasis of hepatoma cells *in vitro* and *in vivo*.

### CEBPA-DT interacts with RNA-binding protein hnRNPC

**Fig. 5:**
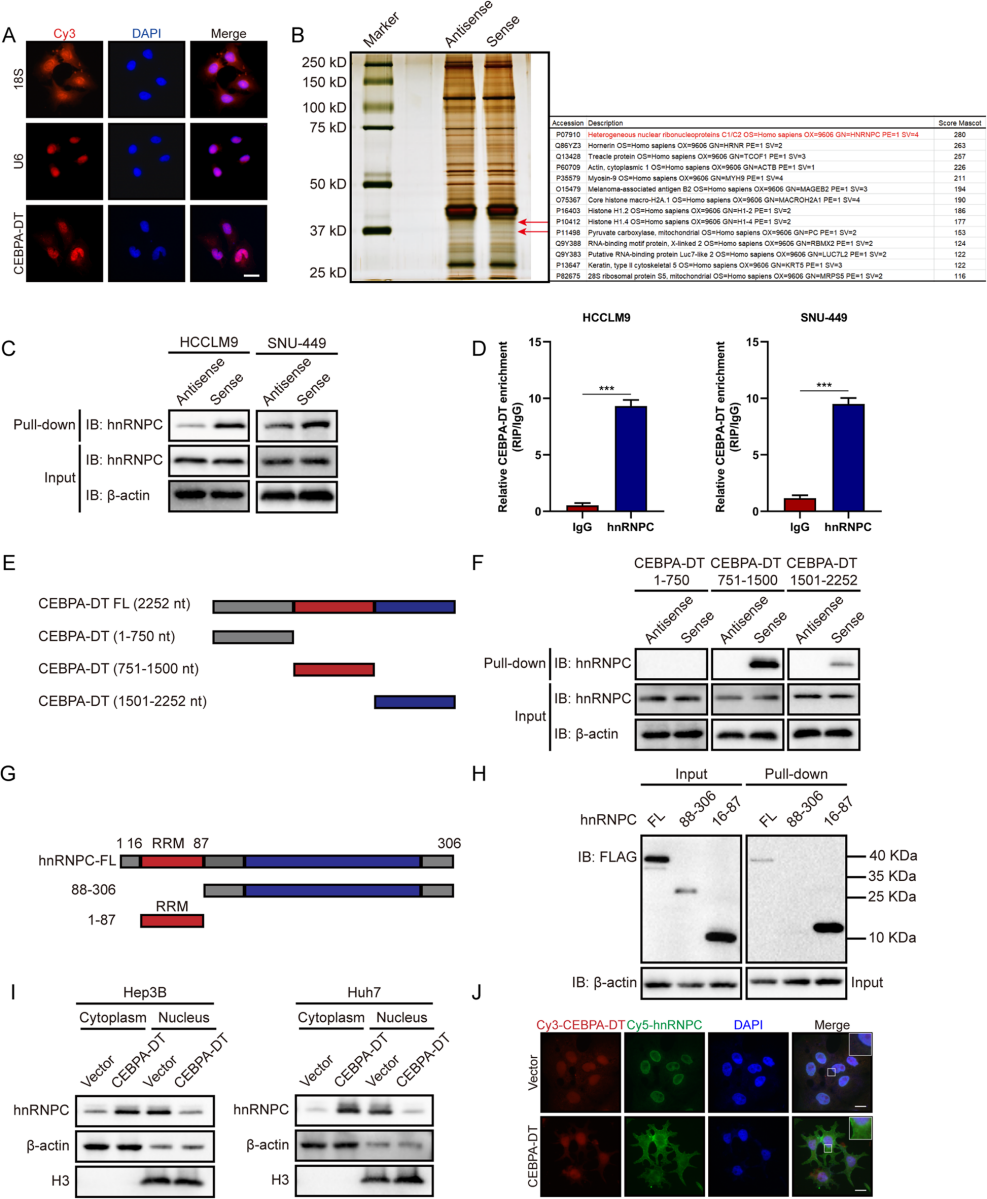
CEBPA-DT interacts with RNA-binding protein hnRNPC. **A** RNA FISH for CEBPA-DT. Nuclei were stained with DAPI. 18S and U6 were applied as positive controls in the cytoplasm and nucleus, respectively. Scale bar, 50 μm. **B** Left: silver staining showed total proteins interact with CEBPA-DT, the specific bands of interest (shown as arrows) were subjected to mass spectrometry. Right: The table showed mass spectrometric results identifying proteins that interacted with CEBPA-DT. **C** Western blot analysis of hnRNPC in protein samples pulled down by CEBPA-DT in indicated HCCLM9 and SNU-449 cells. **D** RIP-qPCR analysis of the enrichment of CEBPA-DT on hnRNPC relative to IgG in indicated HCCLM9 and SNU-449 cells. **E** Diagram of full-length and truncated CEBPA-DT. **F** Western blot analysis of hnRNPC in protein samples pulled down by truncated CEBPA-DT. **G** Diagram of full-length and truncated Flag-hnRNPC. **H** Left: western blot analysis showed the expression of full length (FL) or truncated hnRNPC from lysates of 293 T cells transfected with the indicated plasmids. Right: western blot analysis revealed the proteins pulled-down by CEBPA-DT from the lysates of 293 T cells transfected with the indicated plasmids. **I** Subcellular protein fractionations showed changes of subcellular localization of hnRNPC in indicated Huh7 and Hep3B cells, β-actin and H3 were used as cytoplasmic and nuclear endogenous control, respectively. **J** Co-localization between CEBPA-DT and hnRNPC was observed by FISH and IF in Huh7 cells. Nuclei were stained with DAPI. Scale bar, 20 μm. Date are presented as mean ± SD; *n* = 3. Student’s t test was used. *p < 0.05, **p < 0.01, ***p < 0.001

Multiple studies have highlighted the regulatory roles of lncRNA through sponging miRNAs and binding proteins [9, 25]. To validate whether CEBPA-DT acts as a “miRNA sponge", we conducted the bioinformatic analysis to evaluate the potential binding miRNAs for CEBPA-DT using Starbase [26]. The results suggested that has-miR-532-3p was the target with the most binding possibility. We next established CEBPA-DT wild type and mutation luciferase plasmids (Figure S3A); by co-transfection of the wild type/mutated CEBPA-DT together with NC mimics/ miR-532-3p, relative luciferase activities were measured by Rluc/Fluc. The results showed no significant difference between miR-532-3p group and CEBPA-DT wild-type/mutation group (Figure S3B). Furthermore, the results of RIP assays by using an antibody against argonaute 2 (AGO2) demonstrated that there was no remarkable difference between IgG and AGO2 group (Figure S3C); these observations indicated that CEBPA-DT might not serve as "miRNA sponge". To validate whether CEBPA-DT regulates tumor growth and metastasis via binding with proteins, we next performed biotin-labeled RNA pull-down and mass spectrometry (MS) to screen proteins interacting with CEBPA-DT in hepatoma cells. As RNA FISH and previously performed subcellular RNA fractionation assays proved that CEBPA-DT was predominantly localized in the nucleus (Fig. 5A), only nuclear extracts were applied for RNA pull-down assay. The silver-staining identified a specific protein band at nearly 40 kD and subsequently detected by MS (Fig. 5B). A total of 177 proteins were identified as CEBPA-DT interacting targets. Among them, the RNA binding protein heterogeneous nuclear ribonucleoprotein C (hnRNPC) was with the highest score (Fig. 5B).

To verify the interaction between CEBPA-DT and hnRNPC, we determined the subcellular location of hnRNPC; the results revealed that the hnRNPC mainly existed in the nucleus of hepatoma cells (Figure S3D), consistent with the subcellular location of CEBPA-DT. Next, the biotin-labeled RNA pull-down and RIP assays further confirmed that CEBPA-DT could interact with hnRNPC (Fig. 5C, D). To further validated the specific interacting sites between CEBPA-DT and hnRNPC, we constructed three truncated fragments of CEBPA-DT (1-750nt, 751-1500nt and 1501-2252nt) (Fig. 5E), as well as the truncated hnRNPC fragments based on the RNA recognition motif (RRM, 16-87aa) (Fig. 5G). The RNA-pull down assays revealed that 751–1500 nucleotides were responsible for the interaction between CEBPA-DT and the RNA recognition motif of hnRNPC (Fig. 5F, H). To further clarify the mechanism of the interaction between hnRNPC and CEBPA-DT, we measured the expression levels of hnRNPC in CEBPA-DT control and overexpressing hepatoma cells; we observed that upregulation of CEBPA-DT did not alter the mRNA and protein levels of hnRNPC (Figure S3E, F). A previous study had proved that hnRNPC was partially relocalized from the nucleus to the cytoplasm via interaction with c-*myc* [27]. We hypothesized that the interaction between CEBPA-DT and hnRNPC could activate the subcellular relocalization of hnRNPC. Subcellular protein fractionation assays revealed that overexpression of CEBPA-DT upregulated the protein levels of hnRNPC in the cytoplasm while decreasing that in the nucleus (Fig. 5I). Similar observations were obtained through RNA FISH combined with IF assays (Fig. 5J). Taken together, these data suggesting that CEBPA-DT may physically bind hnRNPC and induce the translocalization of hnRNPC from the nucleus to the cytoplasm of hepatoma cells.

### CEBPA-DT promotes the metastasis in hepatoma cells through the hnRNPC-DDR2 axis

**Fig. 6:**
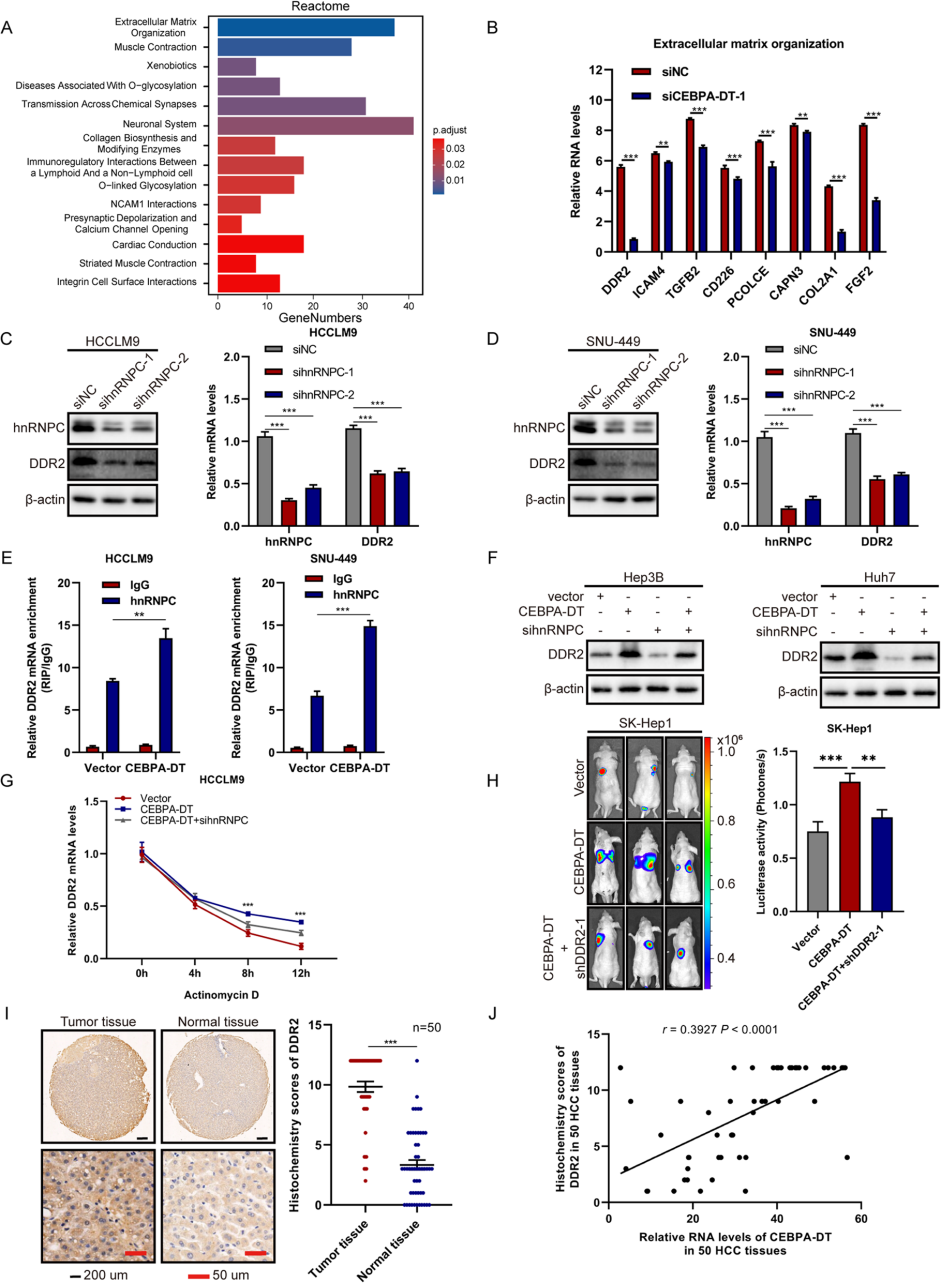
CEBPA-DT promotes the metastasis of hepatoma cells through the hnRNPC-DDR2 axis. **A** Enrichment analysis showed significantly enriched pathways annotated by the Reactome terms ranked based on the significance of the enrichments. **B** Expression levels of 8 down-regulated extracellular matrix-related mRNAs screened by enrichment analysis were measured with RT-qPCR. **C-D** hnRNPC and DDR2 expression levels were detected by western blot and RT-qPCR in hnRNPC silencing cells, respectively. **E** RIP-qPCR analysis of the enrichment of DDR2 mRNA on hnRNPC relative to IgG in indicated control and CEBPA-DT overexpression cells. **F** Western blot analysis of DDR2 expression levels in Hep3B and Huh7 cells co-transfected with CEBPA-DT overexpression vectors, hnRNPC siRNAs or the control vectors. **G** 48 h after transfected with CEBPA-DT overexpression vectors with or without hnRNPC siRNA, DDR2 mRNA levels were detected at different periods after treated with actinomycin D. **H** Representative bioluminescent images and the corresponding statistical analyses of bioluminescent tracking plots for lungs of nude mice injected with stable CEBPA-DT overexpressing SK-Hep1 cells with or without stable DDR2 silencing. **I** IHC staining of DDR2. Left: Representative samples of HCC tissues and adjacent non-tumor tissues. Right: IHC scores of 50 HCC tissues and adjacent non-tumor tissues presented as mean ± SEM, Wilcoxon signed-rank test was used. **J** The correlation between the relative expression of CEBPA-DT and the IHC staining of DDR2 in 50 HCC tissues. The correlation was measured by Pearson correlation analysis. Date are presented as mean ± SD; *n* = 3. Student’s t test was used. *p < 0.05, **p < 0.01, ***p < 0.001

To gain a deeper understanding of the tumorigenic role of CEBPA-DT in hepatoma cells. RNA-sequencing was performed to analyze the change in transcriptome which was affected by the silencing of CEBPA-DT. The cluster analysis revealed a total of 2040 down-regulated genes in CEBPA-DT silencing HCCLM9 cells (Figure S4A). Further enrichment analyses in different databases showed that the extracellular matrix related pathways and genes exhibited the most significant alterations (Fig. 6A, Figure S4B, C, D). The intersection of these analyses revealed 8 down-regulated genes which were subjected to RT-qPCR, the results indicated that the mRNA level of DDR2 was down-regulated most significantly after silencing CEBPA-DT (Fig. 6B).

As a member of the ubiquitously expressed heterogeneous nuclear ribonucleoproteins (hnRNPs) family, hnRNPC serves as RBP to bind nuclear RNAs, and is essential for the stabilization and translation of many mRNAs, typically interacts with the 3’ UTR region of the target mRNA [16, 28, 29]. Considering that CEBPA-DT induced relocalization of hnRNPC from nucleus to cytoplasm, and the DDR2 mRNA level was regulated by CEBPA-DT, we hypothesized that CEBPA-DT might enhance the stability of DDR2 mRNA, thus promoting translation through increasing the cytoplasmic expression of hnRNPC. To verify the conjecture, we measured the mRNA and protein levels of DDR2 after silencing hnRNPC with siRNAs. The results demonstrated that DDR2 mRNA and protein levels were dramatically down-regulated (Fig. 6C, D). More critically, the interactions between hnRNPC and DDR2 mRNA were markedly enhanced after the upregulation of CEBPA-DT (Fig. 6E). Collectively, these results indicated that DDR2 is the downstream target of the CEBPA-DT-hnRNPC complex.

We successively investigated the mechanism of DDR2 down-regulation affected by the CEBPA-DT-hnRNPC axis. The binding potential for hnRNPC and 3' UTR of DDR2 mRNA was predicted by the software of PRIdictor [30]; the results indicated that hnRNPC possessed the high binding potential for 3'UTR of DDR2 mRNA, and the region with the highest binding possibility localized at the 158-163aa of hnRNPC (Figure S4E), which differed from the binding site for CEBPA-DT and hnRNPC, suggesting that there was no competitive binding effect between CEBPA-DT, hnRNPC and DDR2 mRNA. More importantly, by treating the hepatoma cells with actinomycin D, we found that overexpression of CEBPA-DT reduced DDR2 mRNA degradation, and prolonged its half-life, thus increasing the DDR2 mRNA expression, meanwhile deletion of hnRNPC significantly inhibited this promotion, enhanced the DDR2 mRNA level (Fig. 6G). Consistently, the protein levels exhibited the same change trends after upregulation of CEBPA-DT and then silencing of hnRNPC (Fig. 6F).

A previous study has illuminated that DDR2 facilitates HCC invasion and metastasis [31]. Our *in vitro* functional studies suggested that the motility of hepatoma cells was significantly suppressed after silencing the mRNA and protein levels of DDR2 (Figure S5A-F). Functionally, wound-healing and transwell assays showed that overexpression of CEBPA-DT could enhance the motility of hepatoma cells, while deletion of DDR2 could block this enhancement (Figure S6A-D). Similar results were obtained by the lung metastasis model in nude mice (Fig. 6H, Figure S6E, F). Additionally, by applying IHC and RT-qPCR in 50 HCC samples, we found that the protein and mRNA levels of DDR2 were obviously increased in HCC tissues compared to normal tissues (Fig. 6I, Figure S6G). Moreover, the expressions of DDR2 were positively correlated to the expressions of CEBPA-DT in HCC tissues (Fig. 6J). To sum up, the aforementioned data suggested that the CEBPA-DT could promote the metastasis of HCC via the hnRNPC-DDR2 axis.

### CEBPA-DT induces EMT in hepatoma cells

**Fig. 7:**
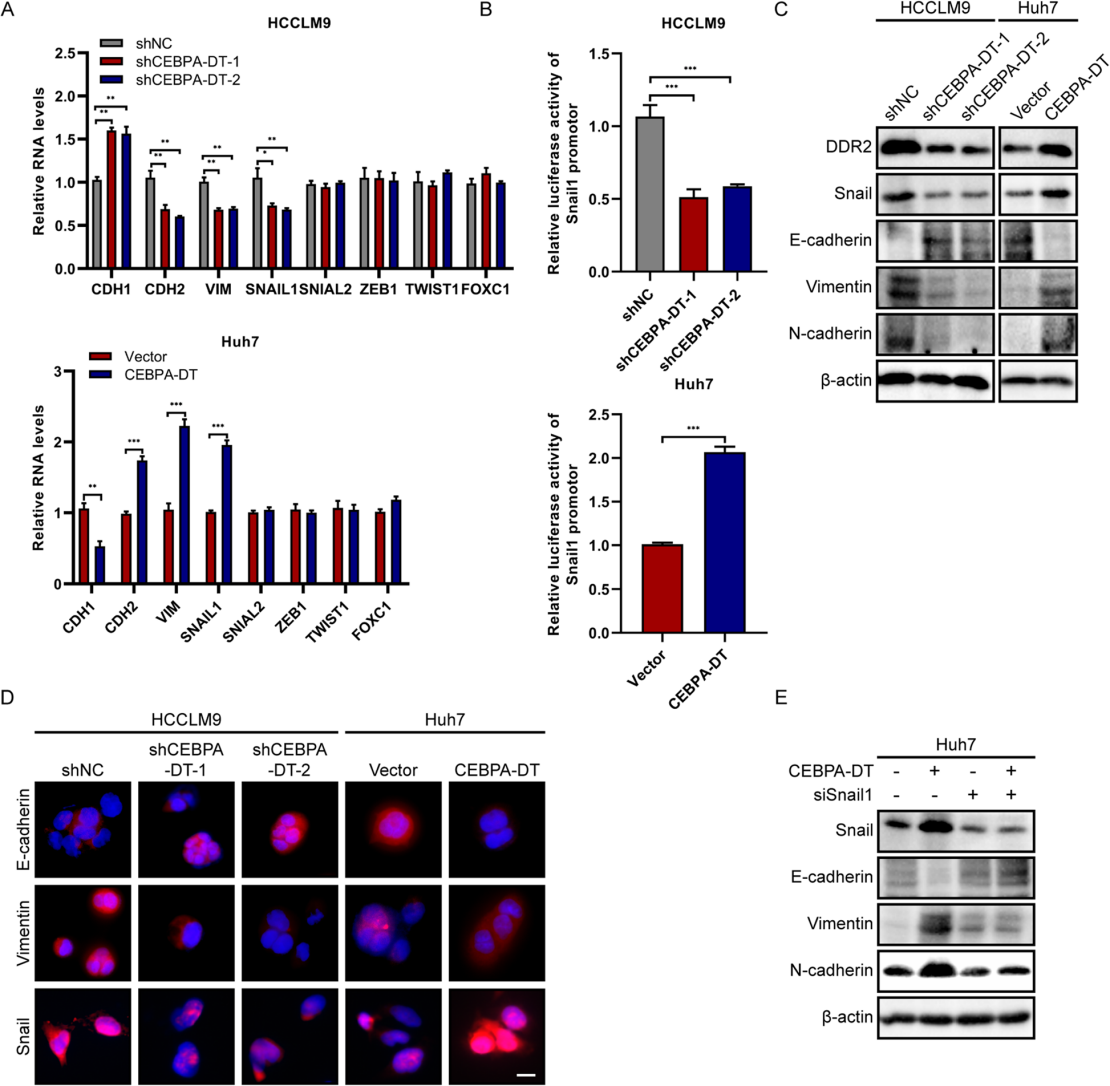
CEBPA-DT induces EMT in hepatoma cells. **A** The expression levels of EMT markers were measured by RT-qPCR in the indicated HCCLM9 and Huh7 cells. **B** Relative luciferase activities of Snail1 promotor were examined by luciferase reporter assays in the indicated HCCLM9 and Huh7 cells. **C** The expression levels of DDR2 and EMT-related proteins Snail, E-cadherin, N-cadherin and Vimentin were measured by western blot assays. **D** The expression levels of EMT-related proteins Snail, E-cadherin and Vimentin were measured by IF assays. Scale bar, 50 μm. **E** The expression levels of EMT-related proteins Snail, E-cadherin, N-cadherin and Vimentin were detected by western blot assays in the indicated cells co-transfected with siSnail1 or negative control. Date are presented as mean ± SD; *n* = 3. Student’s t test was used. *p < 0.05, **p < 0.01, ***p < 0.001

The previous study had demonstrated that CEBPA-DT down-regulation altered the expressions of EMT-related genes [32]; however, the molecular mechanism that CEBPA-DT involves in the EMT process remains unclear. In the present study, we first screened several EMT markers, including epithelial marker E-cadherin (CDH1), mesenchymal markers N-cadherin (CDH2), Vimentin (VIM), SNAIL1, SNAIL2, ZEB1, TWIST1 and FOXC1 by RT-qPCR, the results showed that knockdown of CEBPA-DT induced the expressions of epithelial marker CDH1, while suppressed the expressions of mesenchymal markers (CDH2, VIM, SNAIL1). Meanwhile, overexpression of CEBPA-DT exhibited the opposite results. No significant alteration was observed in ZEB1, SNAIL2, TWIST1 and FOXC1 (Fig. 7A). To examine whether the EMT transcription factor Snail1 was activated by CEBPA-DT, Snail1 promoter luciferase reporter plasmids were transfected into the hepatoma cells. The results demonstrated that CEBPA-DT silencing significantly decreased the activity of the Snail1 promoter, whereas upregulated CEBPA-DT remarkably induced the promoter activity (Fig. 7B). Moreover, the western blot experiments verified that CEBPA-DT overexpression repressed the protein expression of E-cadherin and induced the expressions of N-cadherin, Vimentin, Snail and DDR2 in hepatoma cells, while CEBPA-DT down-regulation showed contrary results (Fig. 7C). In accordance with the western blot assays, the IF experiments revealed that depletion of CEBPA-DT markedly increased the E-cadherin expression and diminished the Vimentin and Snail expressions (Fig. 7D). To further validated that the EMT process was induced via activation of Snail1 by CEBPA-DT, we knocked down Snail1 in Huh7 cells. The promoted expressions for mesenchymal markers by CEBPA-DT upregulation were diminished by silencing of Snail1; opposite trends were observed in the expression of the epithelial marker E-cadherin (Fig. 7E). Taking together, these data suggested that CEBPA-DT induced EMT in hepatoma cells via transcriptional activation of Snail1.

### CEBPA-DT promotes the EMT process via the interaction between DDR2 and β-catenin

**Fig. 8:**
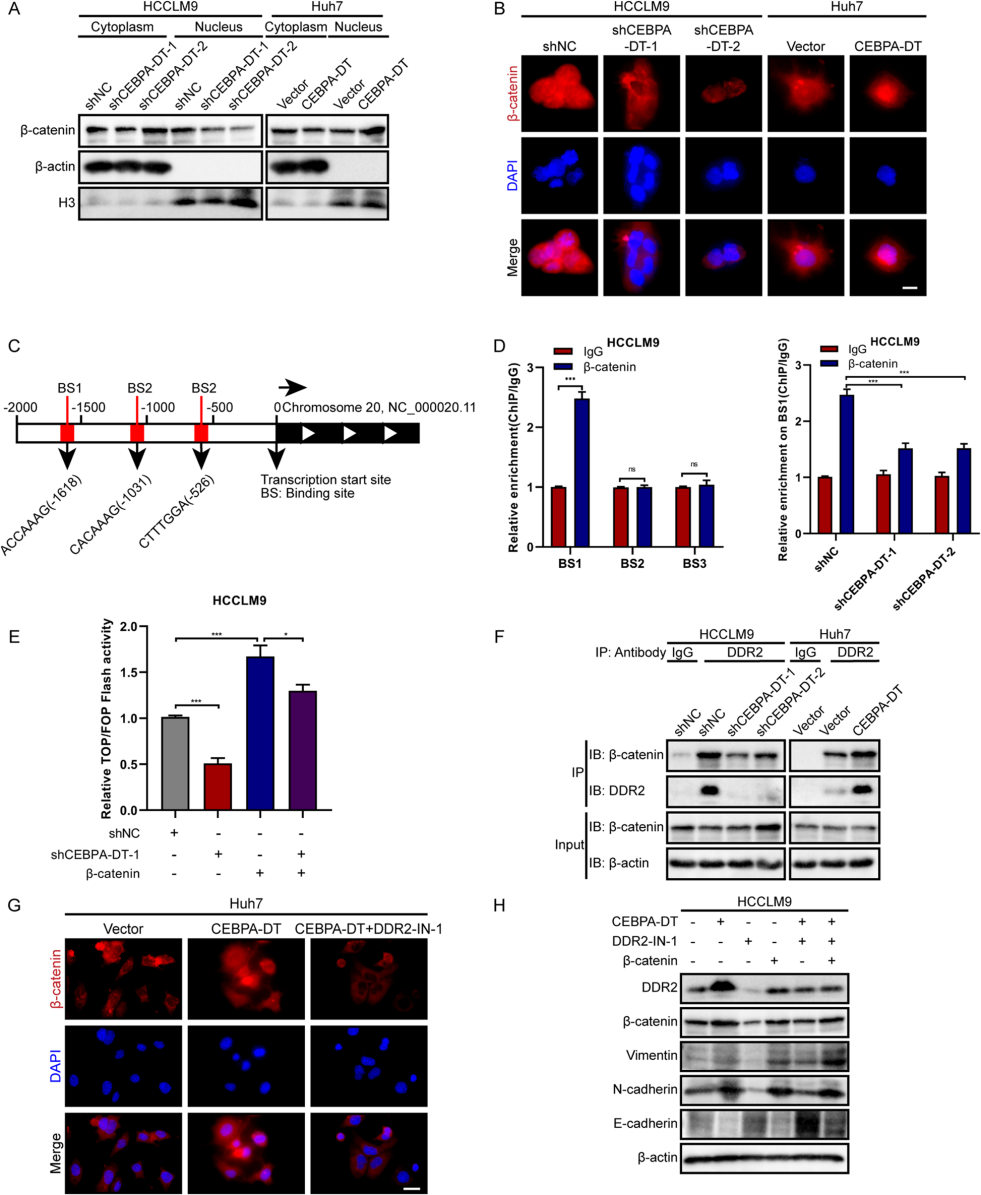
CEBPA-DT promotes the EMT process via the interaction between DDR2 and β-catenin. **A** The expression levels of β-catenin in the nuclear and cytosol in the indicated HCCLM9 and Huh7 were measured by the subcellular fractionation assays, β-actin and H3 were used as the cytoplasmic and nuclear endogenous control. **B** IF assays demonstrated that CEBPA-DT increased the nuclear translocation of β-catenin. Nuclei were stained with DAPI. Scale bar, 50 μm. **C** Schematic diagram of the predicted binding sites of β-catenin on the promoter region of Snail1 gene. **D** Left: ChIP assays showed the enrichment of β-catenin on BSs in the promoter region of Snail1 relative to IgG. Right: ChIP assays showed the enrichment of β-catenin on BSs in the Snail1 promoter region in the indicated cells. **E** TOP/FOP Flash assays showed the transcriptional activities of β-catenin signaling in the indicated HCCLM9 cells. **F** Co-IP assays showed CEBPA-DT regulated the interaction between β-catenin and DDR2 in the indicated cells. **G** IF assays showed CEBPA-DT mediated β-catenin nuclear redistribution was rescued in indicated Huh7 cells treated with DDR2-IN-1. **H** Western blot assays showed CEBPA-DT induced-EMT was regulated by DDR2-β-catenin axis. Date are presented as mean ± SD; *n* = 3. Student’s t test was used. *p < 0.05, **p < 0.01, ***p < 0.001

As the core component of the Wnt/β-catenin pathway, β-catenin has been found to be regularly activated in the metastasis of HCC and intimately linked to the EMT process [33, 34]. Thus, we hypothesized that β-catenin was involved in the CEBPA-DT-regulated EMT process. First, subcellular protein fractionations revealed that β-catenin expression in the nuclear component was significantly upregulated in the CEBPA-DT overexpression hepatoma cells and down-regulated in the CEBPA-DT silencing cells, while the expressions of β-catenin in the cytoplasmic fraction remained unchanged (Fig. 8A). Next, immunofluorescence assays demonstrated that the nuclear β-catenin was markedly relocalized into the cytoplasm in hepatoma cells upon CEBPA-DT silencing. On the contrary, nuclear translocation of β-catenin was promoted after CEBPA-DT upregulation (Fig. 8B). To explore the transcriptional activity of the Wnt/β-catenin signaling, the TOP/FOP-Flash luciferase report assay was performed, which revealed that the transcriptional activity was reduced by knockdown of CEBPA-DT in the HCCLM9-vector cells and in the β-catenin overexpression cells (Fig. 8E).

In order to evaluate the regulatory capacity of β-catenin on Snail1 promoter activity, we conducted the bioinformatic analysis to predict the binding sites of β-catenin on the promoter region of Snail1 (Fig. 8C). Further ChIP-qPCR assays demonstrated significant enrichment of β-catenin on the binding site 1 of the Snial1 promoter region; meanwhile, this enrichment could be markedly repressed by CEBPA-DT silencing (Fig. 8D).

An earlier study had proved the regulatory role of DDR2 in the EMT process [35]. Our data confirmed that DDR2 was regulated by CEBPA-DT. Therefore, we conjectured that there existed an interaction between DDR2 and β-catenin, and this interaction was required for CEBPA-DT regulated EMT process. First, co-Immunoprecipitation assays were conducted using a specific antibody against DDR2, the results showed that DDR2 could indeed interact with β-catenin, and the interaction was weakened upon CEBPA-DT silencing and was strengthened after CEBPA-DT overexpression (Fig. 8F). Next, a specific inhibitor, DDR2-IN-1 was used to repress DDR2. With the presence of DDR2-IN-1, the increased nuclear translocation of β-catenin regulated by overexpression of CEBPA-DT was significantly depleted (Fig. 8G). Finally, the western blot assays revealed that the DDR2 inhibition could lead to the repression of CEBPA-DT induced-EMT process; overexpression of β-catenin rescued this repression (Fig. 8H). In general, the above findings suggested that CEBPA-DT promoted the EMT process via the interaction between DDR2 and β-catenin.

## Discussion

Postoperative metastasis and recurrence remain the major factors that affect the survival of HCC patients [3, 4]. In order to establish efficient approaches to block the metastasis and recurrence of HCC, it is crucial to inspect the molecular mechanisms that drive the biological functions correlated to the metastasis and recurrence of HCC. Emerging studies have reported that lncRNAs play critical roles in regulating tumor metastasis in multiple types of human malignancy. For instance, LncRNA OVM interacts with and stabilizes PPIP5K2 by reducing its ubiquitinated degradation, allowing ovarian cancer cells to secrete complement C5. The complement C5 enriched in TME recruits myeloid-derived suppressor cells (MDSCs), thus promoting metastasis [36]. LncRNA HUMT recruits Y-box binding protein 1 (YBX1) to form a new transcription complex and activates the production of forkhead box k1 (FOXK1), hence increasing VEGF expression (VEGFC) in triple-negative breast cancer (TNBC) [37]. LncRNA BLACAT2 directly interacts with WDR5 and promotes H3K4 methylation, which enhances the expression of VEGF-C epigenetically in bladder cancer with lymph node metastasis [38]. Notwithstanding, the biological functions and underlying mechanisms associated with the metastasis of HCC remain largely uncertain. In the present study, we investigated the differentially expressed lncRNAs in the RNA-seq data of HCC with or without postoperative extrahepatic metastasis. By assessing the abundance of the differentially expressed lncRNAs, a series of candidates were successfully identified, and the most significantly upregulated lncRNA CEBPA-DT was chosen for further study.

Therefore, we discovered the role of CEBPA-DT, also known as CEBPA-AS1 and ADINR, in the progression and metastasis of HCC. Previously, very few studies have illuminated the regulatory role of CEBPA-DT in human cancers and mainly focus on oral squamous cell cancer (OSCC). For example, overexpression of CEBPA-DT could affect the METTL3/METTL14 expression and hence activate downstream BHLHB9. Meanwhile, upregulation of CEBPA-DT could restrain the IL-17 signaling activity, resulting in the breakdown of immune infiltration and cytokine release balance, thus accelerating OSCC growth [39]. Another study showed that CEBPA-DT was upregulated in cisplatin resistance OSCC cells, CEBPA-DT regulated cisplatin chemosensitivity through CEBPA/BCL2-mediated cell apoptosis [40]. For gastrointestinal cancer, the prognostic value of CEBPA-DT was illustrated along with several other lncRNAs (INHBA-AS1, AK001058, UCA1, PPBP, and RGS18) in early gastric cancer [41]. Last but foremost, a recently published paper documented that CEBPA-DT was capable of promoting the EMT process and liver cancer progression [32], but the mechanism underlying the promoting effect was not illuminated. Taken together, these investigations showed that CEBPA-DT was a critical lncRNA in human malignancy, which may serve as an oncogene in liver cancer, indicating that CEBPA-DT is worthy of further investigation. In our present work, we verified that CEBPA-DT was upregulated in HCC tissues compared to non-tumor tissues; high CEBPA-DT expression was relevant to inferior prognosis of HCC patients. Moreover, we assessed the biological function of CEBPA-DT and clarified the molecular mechanisms underlying CEBPA-DT-mediated promotion of metastasis in hepatoma cells. The present study was the first one to illuminate the intrinsic molecular mechanism of CEBPA-DT in liver cancer. The approaches to identify CEBPA-DT were rational by conducting RNA-seq in HCC samples with different clinical features (with or without extrahepatic metastasis), which clearly illustrated the role of CEBPA-DT in HCC metastasis. More importantly, our research was the first to unravel the interacting proteins of CEBPA-DT thoroughly. Therefore, our work will facilitate the understanding of lncRNA function and the implementation of lncRNA as prognostic and therapeutic targets for HCC.

Previous works exhibit that hnRNPC is required for the stabilization and translation of target mRNAs [16, 28, 29], and lncRNA has been reported to be involved in the hnRNPC-mediated mRNA stabilization by interacting with hnRNPC, thus promoting cancer metastasis [16]. It has been reported that hnRNP I translocated to the cytoplasm following the stimulation of pancreatic β-cells, thus promoting insulin production by stabilizing mRNAs encoding proteins that form insulin secretory granules [15]. Similarly, in the present study, interaction between CEBPA-DT and hnRNPC triggered the translocation of hnRNPC to the cytoplasm, stabilized DDR2 mRNA. DDR2 belongs to the receptor tyrosine kinase (RTK) family, mounting evidence has proved DDR2 is correlated to the metastasis of HCC [31, 42], and DDR2 inhibitors have been implicated in the treatment of non-small cell lung cancer (NSCLC) [43]. In our present study, we consistently proved that DDR2 knockdown inhibits metastasis of hepatoma cells. Mechanismly, CEBPA-DT could bind with hnRNPC and induce the interaction between hnRNPC and DDR2 mRNA, reducing the DDR2 mRNA degradation and further promoting metastasis of hepatoma cells. Our future study will concentrate on illuminating the specific bindings sites between hnRNPC and DDR2 mRNA, as well as the underlying mechanism that CEBPAD-T/hnRNPC affects the stabilization of DDR2 mRNA.

Emerging evidence has elucidated the vital role of EMT in the activation of tumor invasion and metastasis, which is a binary course including the transition of tumor cells from epithelial to the mesenchymal condition as well as the development of invasive capability [44, 45]. In the present study, we revealed that the EMT process could be positively regulated by CEBPA-DT, and Snail1 was the essential transcriptional activator involved. β-catenin signaling has been widely reported as a critical regulator in the EMT process via nuclear redistribution of β-catenin [46, 47]. Our work verified CEBPA-DT as an upstream regulator of β-catenin and facilitated the interaction between β-catenin and DDR2, thus inducing nuclear translocation of β-catenin; the activation of β-catenin subsequently binds to the Snail1 promotor to initiate transcription, hence inducing EMT process in hepatoma cells.

## Conclusion

**Fig. 9:**
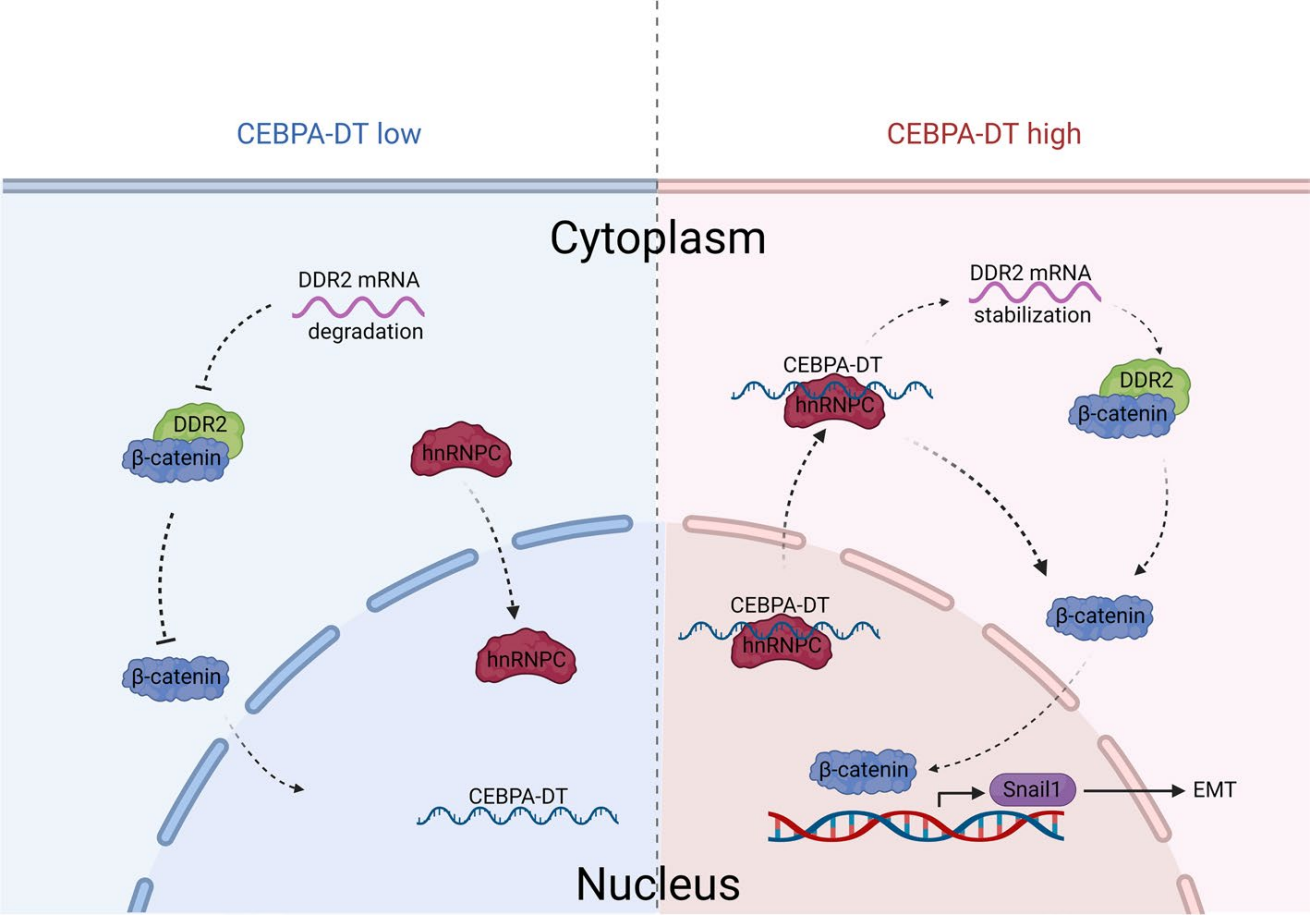
Proposal schematic pathway illustrated the role of CEBPA-DT in the liver cancer metastasis

TO sum up, our study identified CEBPA-DT as an effector of HCC metastasis. Moreover, we revealed that CEBPA-DT promotes progression and metastasis of HCC through activation of DDR2/β-catenin axis via interaction with hnPNPC (Fig. 9). Importantly, our findings offered novel insights into the underlying molecular mechanisms of lncRNA involved-HCC metastasis and laid the foundation for further CEBPA-DT-targeting therapeutical approaches for HCC.

## Supplementary Information

**Additional file 1: Table S1.** Clinical baseline characteristics of 101 patients with HCC according to CEBPA-DT expression level. **Table S2.** Prognostic factors for overall survival and recurrence-free survival by the univariate Cox proportional hazards regression model. **Table S3.** Independent prognostic factors for overall survival and recurrence-free survival by the multivariate Cox proportional hazards regression model. **Table S4.** The antibodies used in this study. **Table S5.** Sequence of primers used for PCR in this study. **Table S6.** Sequence of siRNA and shRNA against specific target in this study.

**Additional file 2: Fig. S1. A **Schematic diagram of the genomic locus of CEBPA-DT (www.ncbi.nlm.nih.gov/). **B **The protein-coding potential of CEBPA-DT predicted by Open reading frame (ORF) Finder software prediction (https://www.ncbi.nlm.nih.gov/orffinder/). **C** The protein-coding potential of CEBPA-DT predicted by Coding-Potential Assessment Tool (wlcb.oit.uci.edu/cpat/).

**Additional file 3: Fig. S2. A** The expression levels of CEBPA-DT in different hepatoma cell lines measured by RT-qPCR. **B** Subcellular location of CEBPA-DT in indicated Huh7 cells examined by subcellular RNA fractionations and RT-qPCR analysis, β-actin and U3 were used as cytoplasmic and nuclear endogenous control, respectively. **C** The expression levels of CEBPA-DT in HCCLM9 and SNU-449 cells transfected with control siRNA or siRNA against CEBPA-DT. **D** qPCR quantification of the expression levels of CEBPA-DT in HCCLM9 and cells transfected with control shRNA or shRNA against CEBPA-DT. **E** qPCR quantification of the expression levels of CEBPA-DT in Hep3B, Huh7 and SK-Hep1 cells stably transfected with lentivirus containing control or CEBPA-DT overexpression vectors. **F** Representative images (fluorescence) of lung metastatic nodules in tail-vein injection models with indicated HCCLM9 and SK-Hep1 cells. Date are presented as mean ± SD; n=3. Student’s t test was used. *p < 0.05, **p < 0.01, ***p < 0.001.

**Additional file 4: Fig. S3. A **Schematic diagram of the binding sites of miR-532-3p on CEBPA-DT. **B** Relative luciferase activities of miR-532-3p binding sites on CEBPA-DT were measured by luciferase reporter assays in indicated 293T cells. **C** RIP-qPCR assays showed the enrichment of CEBPA-DT on AGO2 relative to IgG in indicated HCCLM9 cells. **D** Subcellular localization of hnRNPC in indicated HCCLM9 and SNU-449 cells were measured by subcellular protein fractionations and western blot assays. **E-F** The expression levels of hnRNPC measured by RT-qPCR and western blot in indicated Hep3B and Huh7 cells transfected with control or CEBPA-DT overexpression vectors. Date are presented as mean ± SD; n=3. Student’s t test was used. ns: not significant, *p < 0.05, **p < 0.01, ***p < 0.001.

**Additional file 5: Fig. S4.** **A** Clustering heatmap of significant differentially expressed mRNAs in HCCLM9 cells transfected with negative control or CEBPA-DT siRNAs. **B** Significantly enriched pathways annotated by the Kyoto Encyclopedia of Genes and Genomes (KEGG) database. **C** Significantly enriched pathways annotated by the Gene Ontology (GO) database. **D** Significantly enriched pathways annotated by the Molecular Signatures Database (MSigDB). **E** The binding sites of hnRNPC and 3’UTR of DDR2 were predicted by PRIdictor database (www.rna-society.org).

**Additional file 6: Fig. S5. A-B** The expression levels of DDR2 were measured by western blot and RT-qPCR in indicated HCCLM9 and SNU-449 cells transfected with negative control siRNA or siRNAs targeting DDR2. **C-D** Cell migration capacity of indicated HCCLM9 and SNU-449 cells were measured by scratch wound-healing assays. Scale bar, 100μm. **E-F** Cell migration and invasion capacity of indicated HCCLM9 and SNU-449 cells were measured by transwell migration and matrigel invasion assays. Scale bar, 100μm. Date are presented as mean ± SD; n=3. Student’s t test was used. *p < 0.05, **p < 0.01, ***p < 0.001.

**Additional file 7: Fig. S6. A-B** Scratch wound-healing assays showed cell migration capacity of indicated Hep3B and Huh7 cells stably transfected with lentivirus of shDDR2 vectors and control/ CEBPA-DT overexpressing vectors. Scale bar, 100μm. **C-D** Transwell migration and matrigel invasion assays showed cell migration and invasion capacity of indicated Hep3B and Huh7 cells stably transfected with lentivirus of shDDR2 vectors and control/ CEBPA-DT overexpressing vectors. Scale bar, 100μm. **E** Representative images (fluorescence) of lung metastatic nodules in tail-vein injection models with indicated SK-Hep1 cells. **F** Representative microscopic views of HE staining and the corresponding statistical analyses of lung metastatic nodules (black arrows) in tail-vein injection models with indicated SK-Hep1 cells. Scale bar, 50μm. **G** The expression levels of DDR2 mRNA in 50 HCC tissues and corresponding normal tissues were presented as mean ± SEM, Wilcoxon signed-rank test was used. Date are presented as mean ± SD; n=3. Student’s t test was used. *p < 0.05, **p < 0.01, ***p < 0.001

**Additional file 8: Fig. S7. A-B** Subgroup analyses showed prognostic value of CEBPA-DT for overall survival (top) and recurrence-free survival (bottom) in HCC patients with differential features.

**Additional file 9. **Supplementary RNA sequencing results.

## Data Availability

All data generated or analyzed during this study are included either in this article or in the supplementary information files.

## Abbreviations

AFP: Alpha-fetoprotein
AGO2: Argonaute 2
CCK-8: Cell counting kit-8
CEBPA-DT: CCAAT Enhancer Binding Protein Alpha- divergent transcript
ChIP: Chromatin immunoprecipitation
Co-IP: Co-immunoprecipitation
DDR2: Discoidin domain-containing receptor 2
EMT: Epithelial-mesenchymal transition
FC: Fold change
FDR: False discovery rate
FISH: RNA fluorescence in situ hybridization
GFP: Green florescence protein
HCC: Hepatocellular carcinoma
HE staining: Hematoxylin and eosin staining
hnRNPC: Heterogeneous nuclear ribonucleoprotein C
IF: Immunofluorescence
IHC: Immunohistochemistry
LncRNA: Long non-coding RNA
MS: Mass spectrometry
MVI: Microvascular invasion
NCBI: National Center for Biotechnology Information
OS: Overall survival
PLC: Primary liver cancer
RFS: Recurrence-free survival
RIP: RNA immunoprecipitation
RRM: RNA recognition motif
RTK: Receptor tyrosine kinase
RT-qPCR: Real-time quantitative polymerase chain reaction
siRNA: Small interfering RNA
TCGA: The Cancer Genome Atlas
UTR: Untranslated region
