# Supplementary material for: LncRNA CEBPA-DT promotes liver cancer metastasis through DDR2/β-catenin activation via interacting with hnRNPC
Source: J Exp Clin Cancer Res. 2022 Dec 6;41:335. doi: 10.1186/s13046-022-02544-6 (PMC9724427; doi:10.1186/s13046-022-02544-6)
Supplement: Supplementary file 1 — Additional file 1: Table S1. Clinical baseline characteristics of 101 patients with HCC according to CEBPA-DT expression level. Table S2. Prognostic factors for overall survival and recurrence-free survival by the univariate Cox proportional hazards regression model. Table S3. Independent prognostic factors for overall survival and recurrence-free survival by the multivariate Cox proportional hazards regression model. Table S4. The antibodies used in this study. Table S5. Sequence of primers used for PCR in this study. Table S6. Sequence of siRNA and shRNA against specific target in this study. [file 13046_2022_2544_MOESM1_ESM.docx]

Table S1. Clinical baseline characteristics of 101 patients with HCC according to
CEBPA-DT expression level.

| Characteristics | CEBPA-DT | | P value |
| --- | --- | --- | --- |
|  | Low (n=44) | High (n=44) |  |
| Age, years, mean ± SD | 51.5 ± 11.1 | 50.2 ± 11.8 | 0.592 |
| Gender, male, n (%) | 38 (86.4%) | 35 (79.5%) | 0.395 |
| HBsAg, positive, n (%) | 41 (93.2%) | 32 (72.7%) | 0.011 |
| AFP, ng/μL, mean ± SD | 209.1 ± 396.9 | 597.0 ± 548.2 | <0.001 |
| Tumor size, cm, mean ± SD | 3.7 ± 1.9 | 6.7 ± 3.3 | <0.001 |
| Multiple tumor nodules, n (%) | 10 (22.7%) | 8 (18.2%) | 0.597 |
| Poor tumor differentiation, n (%) | 11 (25.0%) | 24 (54.5%) | 0.005 |
| Microvascular invasion, n (%) | 4 (9.1%) | 20 (45.5%) | <0.001 |
| BCLC stage (B-C), n (%) | 15 (34.1%) | 32 (72.7%) | <0.001 |
| Overall survival months, mean ± SD | 40.0 ± 17.8 | 31.0 ± 20.4 | 0.03 |
| Recurrence-free survival months. mean ± SD | 26.6 ± 15.2 | 19.1 ± 18.9 | 0.043 |

Chi-square test was used to test the association between two categorical variables. AFP,
alpha-fetoprotein; BCLC Barcelona Clinic Liver Cancer; HBsAg, hepatitis B surface
antigen.

Table S2. Prognostic factors for overall survival and recurrence-free survival by
the univariate Cox proportional hazards regression model.

| Characteristics | Overall survival | | | Recurrence-free survival | | | |
| --- | --- | --- | --- | --- | --- | --- | --- |
|  | HR | 95% CI | P value | HR | 95% CI | P value |  |
| Gender, Female/Male | 0.858 | 0.194-3.779 | 0.839 | 1.67 | 0.856-3.257 | 0.132 |  |
| Age, >60/≤60 | 1.785 | 0.619-5.149 | 0.283 | 1.088 | 0.559-2.116 | 0.803 |  |
| HBsAg, Positive/Negative | 1.191 | 0.27-5.241 | 0.817 | 0.484 | 0.232-1.008 | 0.052 |  |
| Tumor differentiation, Poor/Well-Moderate | 1.696 | 0.636-4.521 | 0.001 | 0.291 | 0.808-2.405 | 0.231 |  |
| Tumor size, >5cm/≤5cm | 2.331 | 0.862-6.3 | 0.095 | 1.893 | 1.102-3.253 | 0.021 |  |
| Tumor number, Multiple/Solitary | 0.715 | 0.164-3.102 | 0.654 | 1.175 | 0.628-2.199 | 0.612 |  |
| Microvascular invasion, With/Without | 7.246 | 2.618-20.051 | <0.001 | 2.732 | 1.532-4.873 | <0.001 |  |
| AFP, >400ng/μL/≤400ng/μL | 2.156 | 0.801-5.802 | 0.128 | 2.207 | 0.685-4.126 | 0.113 |  |
| BCLC stage, B-C/0-A | 3.952 | 1.266-12.338 | 0.018 | 1.929 | 1.113-3.343 | 0.019 |  |
| Distant metastasis, With/Without | 3.07 | 0.872-10.804 | 0.081 | 1.636 | 0.697-3.836 | 0.157 |  |
| CEBPA-DT, High/Low | 2.127 | 0.772-5.857 | 0.044 | 1.528 | 0.889-2.626 | 0.025 |  |

HR, hazard ratio; CI, confidence interval; AFP, alpha-fetoprotein; BCLC Barcelona Clinic Liver Cancer; HBsAg, hepatitis B surface antigen.

Table S3. Independent prognostic factors for overall survival and recurrence-free
survival by the multivariate Cox proportional hazards regression model.

| Characteristics | Overall survival | | | Recurrence-free survival | | |
| --- | --- | --- | --- | --- | --- | --- |
|  | HR | 95% CI | P value | HR | 95% CI | P value |
| HBsAg, Positive/Negative |  |  |  | 1.436 | 0.69-3.012 | 0.05 |
| Tumor differentiation, Poor/Well-Moderate | 1.3397 | 0.223-2.488 | 0.634 |  |  |  |
| Tumor size, >5cm/≤5cm | 1.1445 | 0.208-3.663 | 0.853 | 1.233 | 0.441-3.389 | 0.683 |
| Microvascular invasion, With/Without | 9.924 | 2.409-40.879 | 0.001 | 2.856 | 1.467-5.56 | 0.002 |
| AFP, >400ng/μL/≤400ng/μL | 2.167 | 0.672-6.979 | 0.094 | 1.417 | 0.372-5.334 | 0.283 |
| BCLC stage, B-C/0-A | 2.812 | 0.524-15.065 | 0.027 | 2.204 | 0.412-5.51 | 0.056 |
| Distant metastasis, With/Without | 3.942 | 0.824-18.85 | 0.085 | 1.205 | 0.462-3.14 | 0.072 |
| CEBPA-DT, High/Low | 2.443 | 0.083-8.006 | 0.007 | 2.947 | 0.492-4.823 | 0.008 |

HR, hazard ratio; CI, confidence interval; AFP, alpha-fetoprotein; BCLC Barcelona Clinic Liver Cancer; HBsAg, hepatitis B surface antigen.

Table S4. The antibodies used in this study

| Antibody | Supplier | Catalogue number |
| --- | --- | --- |
| hnRNPC | HUABIO | ET1611-2 |
| DDR2 | CST | 12133 |
| DDR2 | Santa Cruz | sc-81707 |
| β-actin | Zenbio | T200068-8F10 |
| H3 | Abclonal | A2348 |
| Ki67 | Servicebio | GB13030-2 |
| Snail | Abclonal | A5243 |
| E-cadherin | Abclonal | A3044 |
| N-cadherin | Santa Cruz | sc-59987 |
| Vimentin | Santa Cruz | sc-6260 |
| β-catenin | CST | 8480 |

Table S5. Sequence of primers used for PCR in this study

| Primer | Sequences |
| --- | --- |
| CEBPA-DT forward | 5'-GGGCACAGCTAAAAACACCG-3' |
| CEBPA-DT reverse | 5'-GAGCTCTGGACCGAAAACGA-3' |
| U6 forward | 5’-GCTTCGGCAGCACATATACTAAAAT-3’ |
| U6 reverse | 5’- CGCTTCACGAATTTGCGTGTCAT -3’ |
| U3 forward | 5'- TTCTCTGAGCGTGTAGAGCACCGA -3' |
| U3 reverse | 5'- GATCATCAATGGCTGACGGCAGTT -3' |
| β-actin forward | 5'- GGGAAATCGTGCGTGACATTAAG-3' |
| β-actin reverse | 5'- TGTGTTGGCGTACAGGTCTTTG-3' |
| ICAM4 forward | 5'-TGATTTTGGAGCCTCCGGTC-3' |
| ICAM4 reverse | 5'- TAGGTCAAGGTCACGTTGGC-3' |
| TGFB2 forward | 5'-GCGCTACATCGACAGCAAAG-3' |
| TGFB2 reverse | 5'- TGCAGCAGGGACAGTGTAAG-3' |
| CD226 forward | 5'-AGCTTTGGGCAAGGGCTATT-3' |
| CD226 reverse | 5'-TGCATGAGAGTGAGGCCAAG-3' |
| PCOLCE forward | 5'-GCTACGATGCTCTGGAGGTC-3' |
| PCOLCE reverse | 5'-CATCCGTCGTCATCCTCAGG-3' |
| CAPN3 forward | 5'-CGATGACCCTGATGACTCGG-3' |
| CAPN3 reverse | 5'-GAAGTCCTTCTGCAGGTGCT-3' |
| COL2A1 forward | 5'-GAGCCAAAGGATCTGCTGGT-3' |
| COL2A1 reverse | 5'-TTGGGGCCTTGTTCACCTTT-3' |
| FGF2 forward | 5'-GCTGTACTGCAAAAACGGGG-3' |
| FGF2 reverse | 5'-AGCCAGGTAACGGTTAGCAC-3' |
| hnRNPC forward | 5'-CAGAACACCCTTCTCCGTCC-3' |
| hnRNPC reverse | 5'-GAGGAGGAGGAGGTACACGT-3' |
| DDR2 forward | 5'-CTGGCTTCCTGCAAACACAC-3' |
| DDR2 reverse | 5'-AGGAAGAGTGCACCACATCG-3' |
| CDH1 forward | 5'-AGCCCCGCCTTATGATTCTCTG-3' |
| CDH1 reverse | 5'-TGCCCCATTCGTTCAAGTAGTCAT-3' |
| CDH2 forward | 5'-AGTGGCAGCTGGACTTGATC-3' |
| CDH2 reverse | 5'-CCGTGGCTGTGTTTGAAAGG-3' |
| VIM forward | 5'-TTGAACGCAAAGTGGAATC-3' |
| VIM reverse | 5'-AGGTCAGGCTTGGAAACA-3' |
| SNAIL1 forward | 5'-GACCCCAATCGGAAGCCTAACTAC-3' |
| SNAIL1 reverse | 5'-AGCCTTTCCCACTGTCCTCATC-3' |
| SNAIL2 forward | 5'-CCTCCATCTGACACCTCC-3' |
| SNAIL2 reverse | 5'-CCCAGGCTCACATATTCC-3' |
| ZEB1 forward | 5'-AAGTGGCGGTAGATGGTA-3' |
| ZEB1 reverse | 5'-TTGTAGCGACTGGATTTT-3' |
| TWIST1 forward | 5'-CTCAAGAGGTCGTGCCAATC-3' |
| TWIST1 reverse | 5'-CCCAGTATTTTTATTTCTAAAGGTGTT-3' |
| FOXC1 forward | 5'-CAGCATCCGCCACAACCTCT-3' |
| FOXC1 reverse | 5'-GCAGCCTGTCCTTCTCCTCCT-3' |
| BS1 forward | 5'-GGTGCATATGAAAAGTTGCTCCA-3' |
| BS1 reverse | 5'-TCCTTCATCCTCACCAGCAC-3' |
| BS2 forward | 5'-ACGACACACTCACAAAGACA-3' |
| BS2 reverse | 5'-AGCTCCCTAGTTCTGCCCTT-3' |
| BS3 forward | 5'-GGAACCATCTGAAGGGGTGG-3' |
| BS3 reverse | 5'-TCCAGGCTGCAGTTTCTACG-3' |

Table S6. Sequence of siRNA and shRNA against specific target in this study

| siRNA names | Sequences |
| --- | --- |
| shCEBPA-DT-1 sense | 5’-CAACAUAGUCCCAGUGAUUtt-3’ |
| shCEBPA-DT-1 anti-sense | 5’-AAUCACUGGGACUAUGUUGtt-3’ |
| shCEBPA-DT-2 sense | 5’-AUUCAACAUAGUCCCAGUGtt-3’ |
| shCEBPA-DT-2 anti-sense | 5’-CACUGGGACUAUGUUGAAUtt-3’ |
| shDDR2-1 sense | 5’-GACUUCAGAUGCUGUAAGUUAUUtt-3’ |
| shDDR2-1 anti-sense | 5’-AAUAACUUACAGCAUCUGAAGUCtt-3’ |
| shDDR2-2 sense | 5’-GAGUCUUCUUAAUAAGUUGAUCUtt-3’ |
| shDDR2-2 anti-sense | 5’-AGAUCAACUUAUUAAGAAGACUCtt-3’ |
| sihnRNPC sense | 5’-UUCGAAGUAUGGCAAAAUUGUGGtt-3’ |
| sihnRNPC anti-sense | 5’-CCACAAUUUUGCCAUACUUCGAAtt-3’ |
| siSnail1 sense | 5’-AGGACUCUAAUCCAGAGUUUACCtt-3’ |
| siSnail1 anti-sense | 5’-GGUAAACUCUGGAUUAGAGUCCUtt-3’ |
