# Supplementary figures and images for: LncRNA CEBPA-DT promotes liver cancer metastasis through DDR2/β-catenin activation via interacting with hnRNPC
Source: J Exp Clin Cancer Res. 2022 Dec 6;41:335. doi: 10.1186/s13046-022-02544-6 (PMC9724427; doi:10.1186/s13046-022-02544-6)

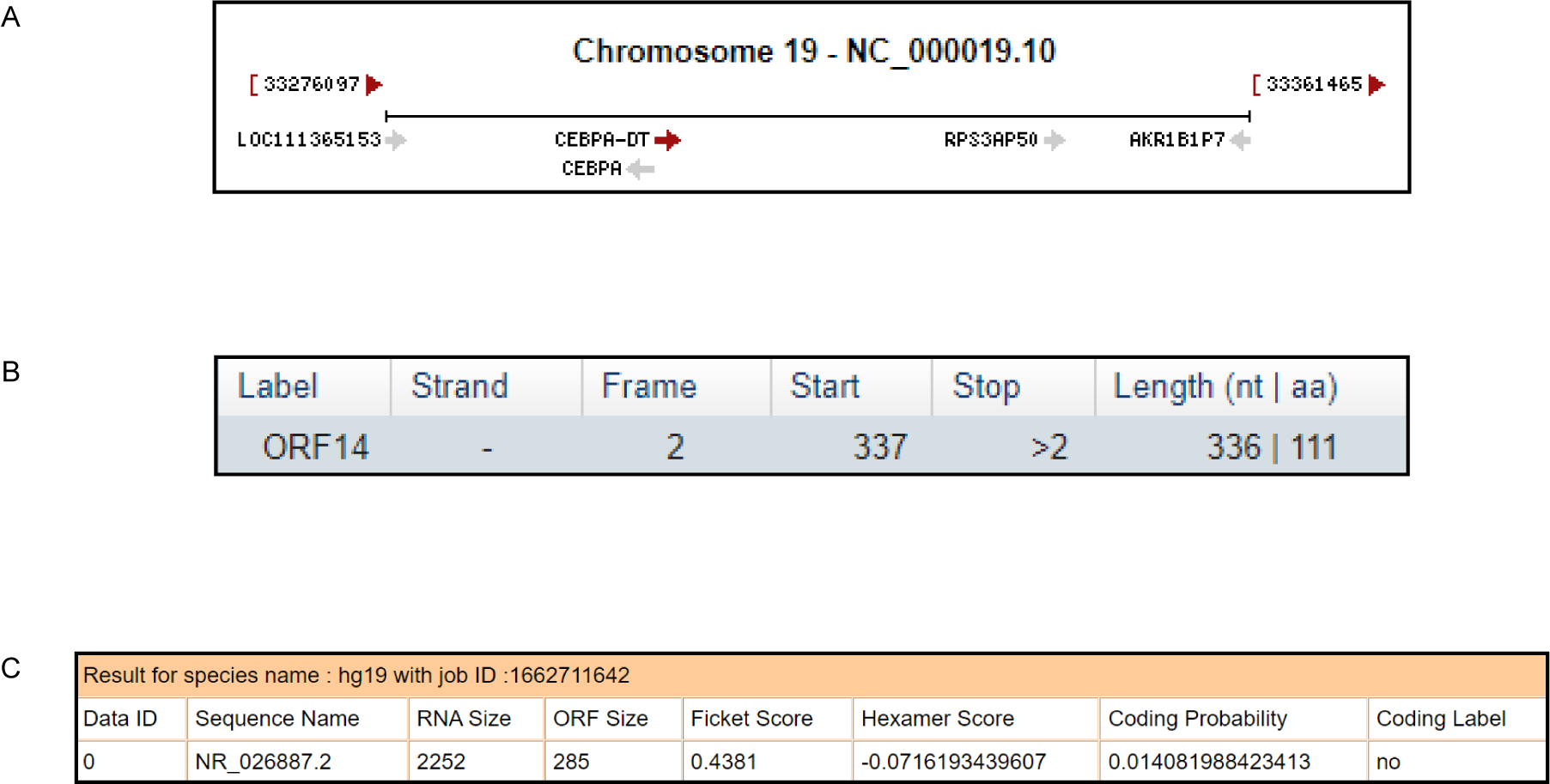

Supplement: Supplementary file 2 — Additional file 2: Fig. S1. A Schematic diagram of the genomic locus of CEBPA-DT (www.ncbi.nlm.nih.gov/). B The protein-coding potential of CEBPA-DT predicted by Open reading frame (ORF) Finder software prediction (https://www.ncbi.nlm.nih.gov/orffinder/). C The protein-coding potential of CEBPA-DT predicted by Coding-Potential Assessment Tool (wlcb.oit.uci.edu/cpat/). [file 13046_2022_2544_MOESM2_ESM.tif]

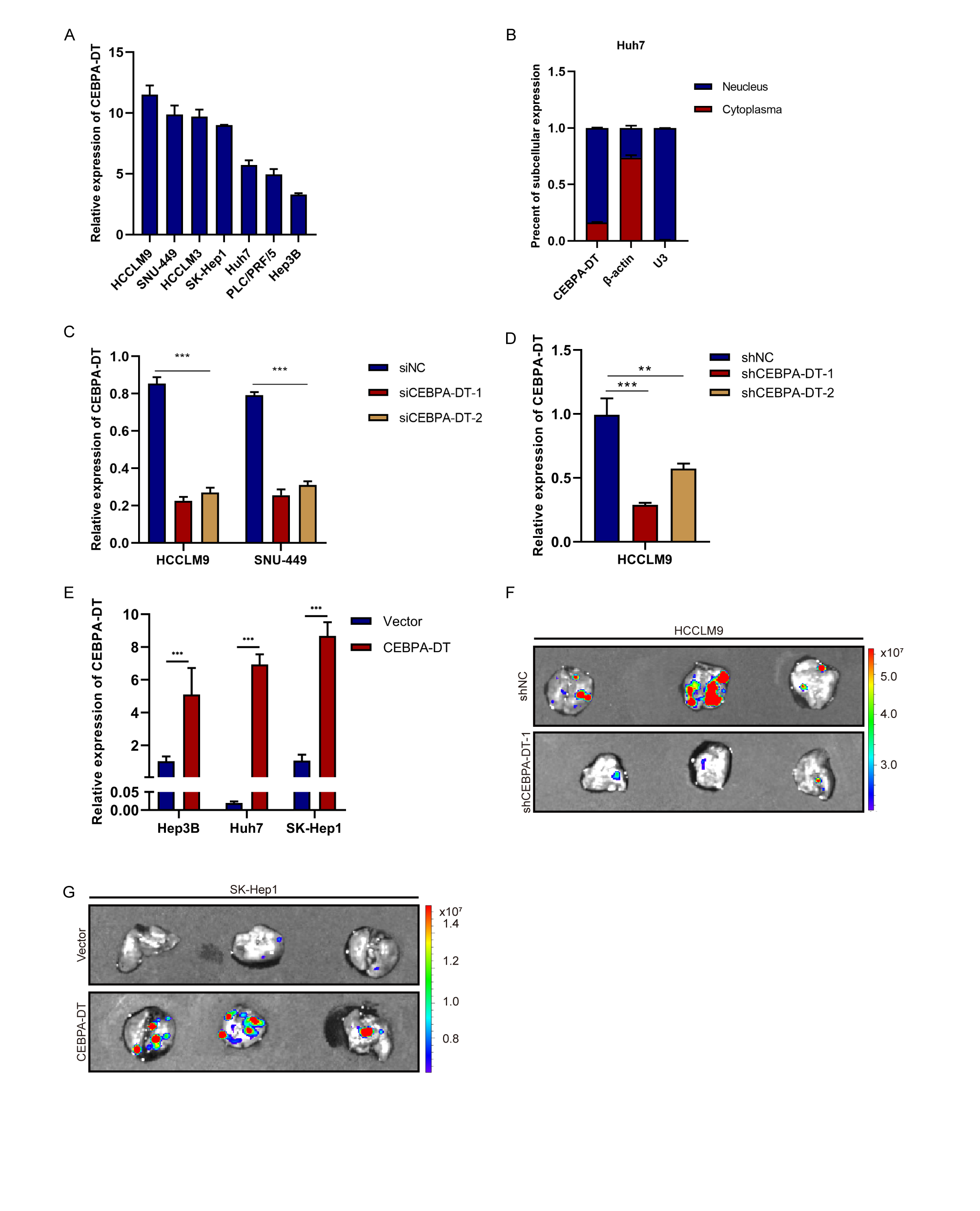

Supplement: Supplementary file 3 — Additional file 3: Fig. S2. A The expression levels of CEBPA-DT in different hepatoma cell lines measured by RT-qPCR. B Subcellular location of CEBPA-DT in indicated Huh7 cells examined by subcellular RNA fractionations and RT-qPCR analysis, β-actin and U3 were used as cytoplasmic and nuclear endogenous control, respectively. C The expression levels of CEBPA-DT in HCCLM9 and SNU-449 cells transfected with control siRNA or siRNA against CEBPA-DT. D qPCR quantification of the expression levels of CEBPA-DT in HCCLM9 and cells transfected with control shRNA or shRNA against CEBPA-DT. E qPCR quantification of the expression levels of CEBPA-DT in Hep3B, Huh7 and SK-Hep1 cells stably transfected with lentivirus containing control or CEBPA-DT overexpression vectors. F Representative images (fluorescence) of lung metastatic nodules in tail-vein injection models with indicated HCCLM9 and SK-Hep1 cells. Date are presented as mean ± SD; n=3. Student’s t test was used. *p < 0.05, **p < 0.01, ***p < 0.001. [file 13046_2022_2544_MOESM3_ESM.tif]

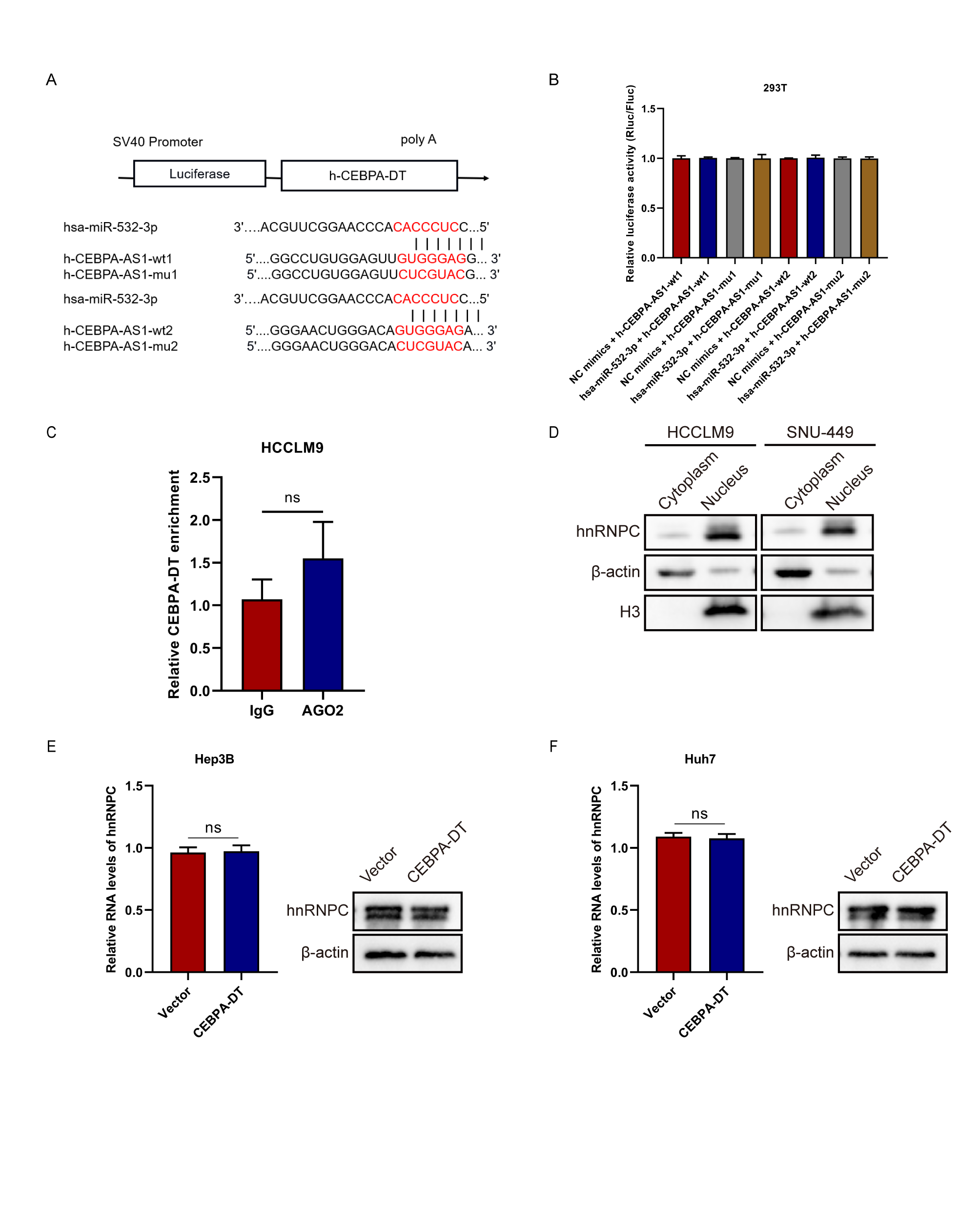

Supplement: Supplementary file 4 — Additional file 4: Fig. S3. A Schematic diagram of the binding sites of miR-532-3p on CEBPA-DT. B Relative luciferase activities of miR-532-3p binding sites on CEBPA-DT were measured by luciferase reporter assays in indicated 293T cells. C RIP-qPCR assays showed the enrichment of CEBPA-DT on AGO2 relative to IgG in indicated HCCLM9 cells. D Subcellular localization of hnRNPC in indicated HCCLM9 and SNU-449 cells were measured by subcellular protein fractionations and western blot assays. E-F The expression levels of hnRNPC measured by RT-qPCR and western blot in indicated Hep3B and Huh7 cells transfected with control or CEBPA-DT overexpression vectors. Date are presented as mean ± SD; n=3. Student’s t test was used. ns: not significant, *p < 0.05, **p < 0.01, ***p < 0.001. [file 13046_2022_2544_MOESM4_ESM.tif]

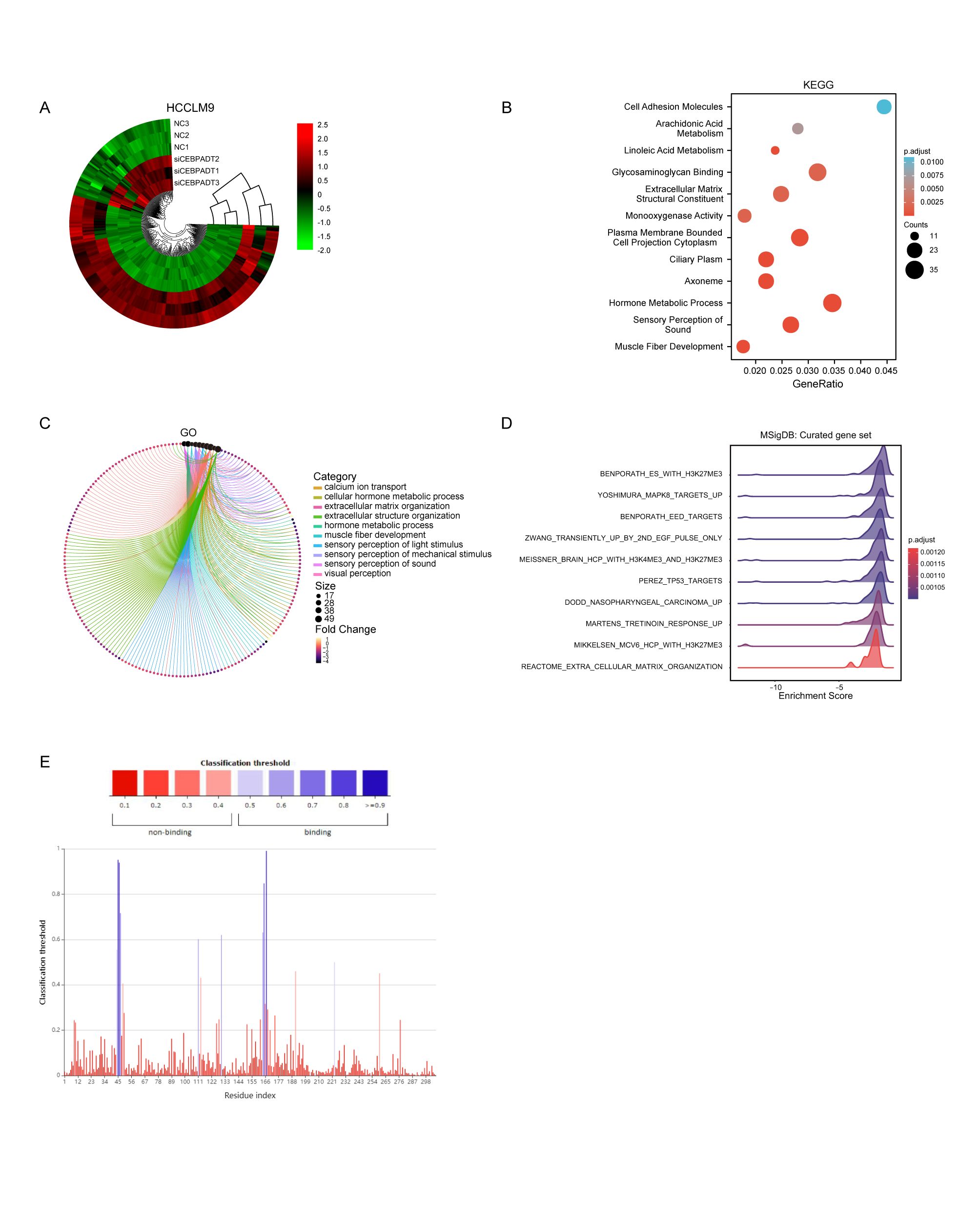

Supplement: Supplementary file 5 — Additional file 5: Fig. S4. A Clustering heatmap of significant differentially expressed mRNAs in HCCLM9 cells transfected with negative control or CEBPA-DT siRNAs. B Significantly enriched pathways annotated by the Kyoto Encyclopedia of Genes and Genomes (KEGG) database. C Significantly enriched pathways annotated by the Gene Ontology (GO) database. D Significantly enriched pathways annotated by the Molecular Signatures Database (MSigDB). E The binding sites of hnRNPC and 3’UTR of DDR2 were predicted by PRIdictor database (www.rna-society.org). [file 13046_2022_2544_MOESM5_ESM.tif]

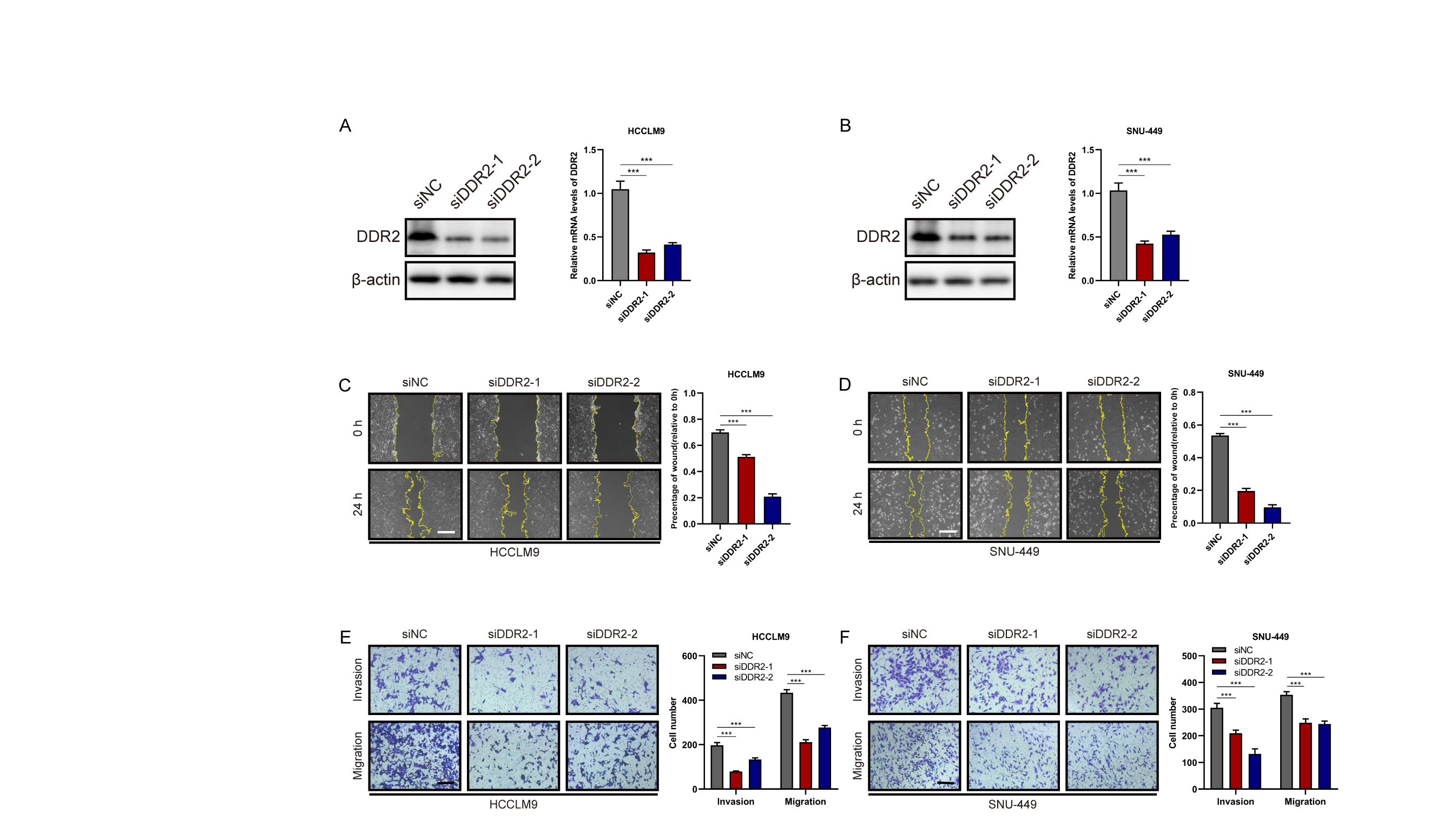

Supplement: Supplementary file 6 — Additional file 6: Fig. S5. A-B The expression levels of DDR2 were measured by western blot and RT-qPCR in indicated HCCLM9 and SNU-449 cells transfected with negative control siRNA or siRNAs targeting DDR2. C-D Cell migration capacity of indicated HCCLM9 and SNU-449 cells were measured by scratch wound-healing assays. Scale bar, 100μm. E-F Cell migration and invasion capacity of indicated HCCLM9 and SNU-449 cells were measured by transwell migration and matrigel invasion assays. Scale bar, 100μm. Date are presented as mean ± SD; n=3. Student’s t test was used. *p < 0.05, **p < 0.01, ***p < 0.001. [file 13046_2022_2544_MOESM6_ESM.tif]

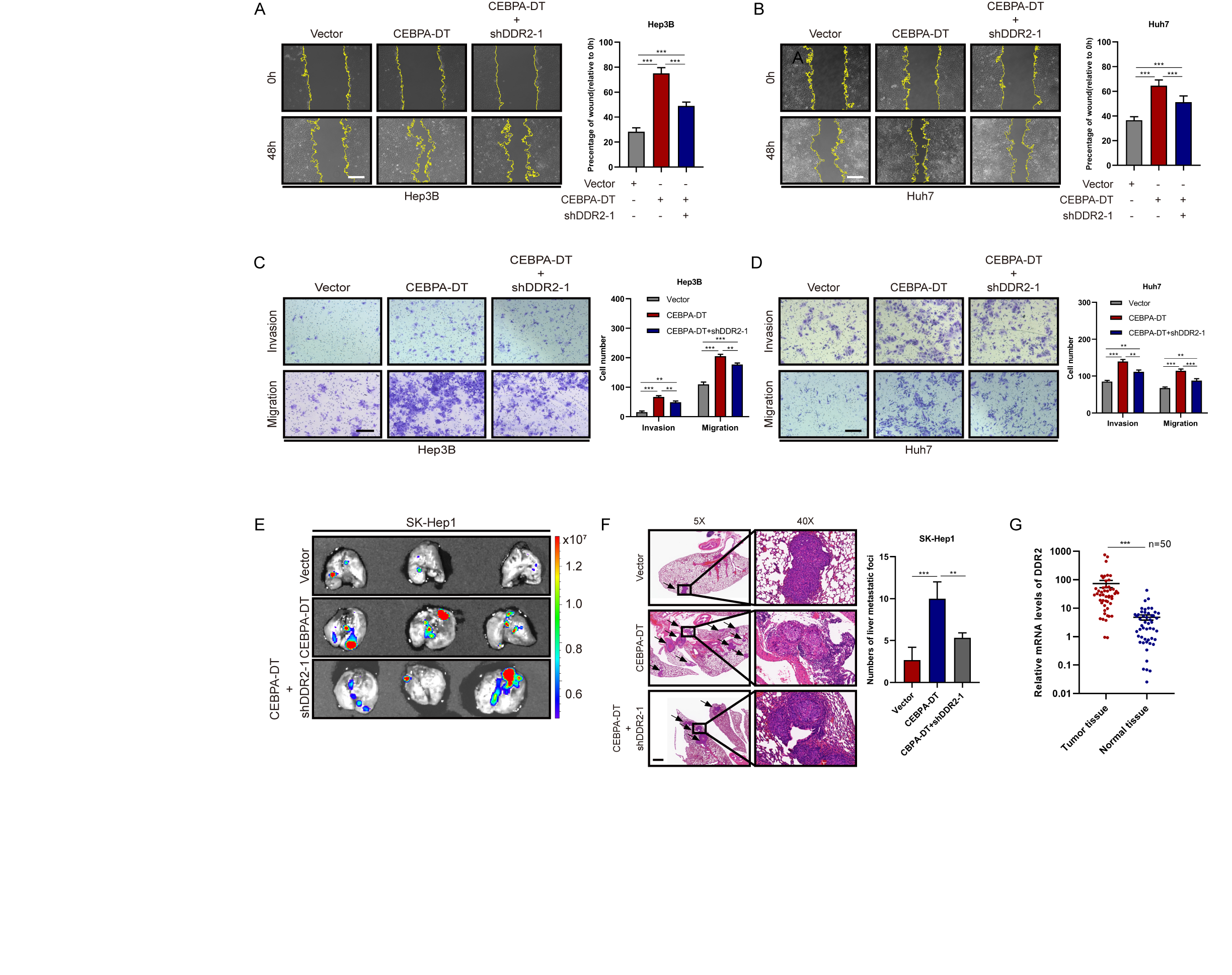

Supplement: Supplementary file 7 — Additional file 7: Fig. S6. A-B Scratch wound-healing assays showed cell migration capacity of indicated Hep3B and Huh7 cells stably transfected with lentivirus of shDDR2 vectors and control/ CEBPA-DT overexpressing vectors. Scale bar, 100μm. C-D Transwell migration and matrigel invasion assays showed cell migration and invasion capacity of indicated Hep3B and Huh7 cells stably transfected with lentivirus of shDDR2 vectors and control/ CEBPA-DT overexpressing vectors. Scale bar, 100μm. E Representative images (fluorescence) of lung metastatic nodules in tail-vein injection models with indicated SK-Hep1 cells. F Representative microscopic views of HE staining and the corresponding statistical analyses of lung metastatic nodules (black arrows) in tail-vein injection models with indicated SK-Hep1 cells. Scale bar, 50μm. G The expression levels of DDR2 mRNA in 50 HCC tissues and corresponding normal tissues were presented as mean ± SEM, Wilcoxon signed-rank test was used. Date are presented as mean ± SD; n=3. Student’s t test was used. *p < 0.05, **p < 0.01, ***p < 0.001 [file 13046_2022_2544_MOESM7_ESM.tif]

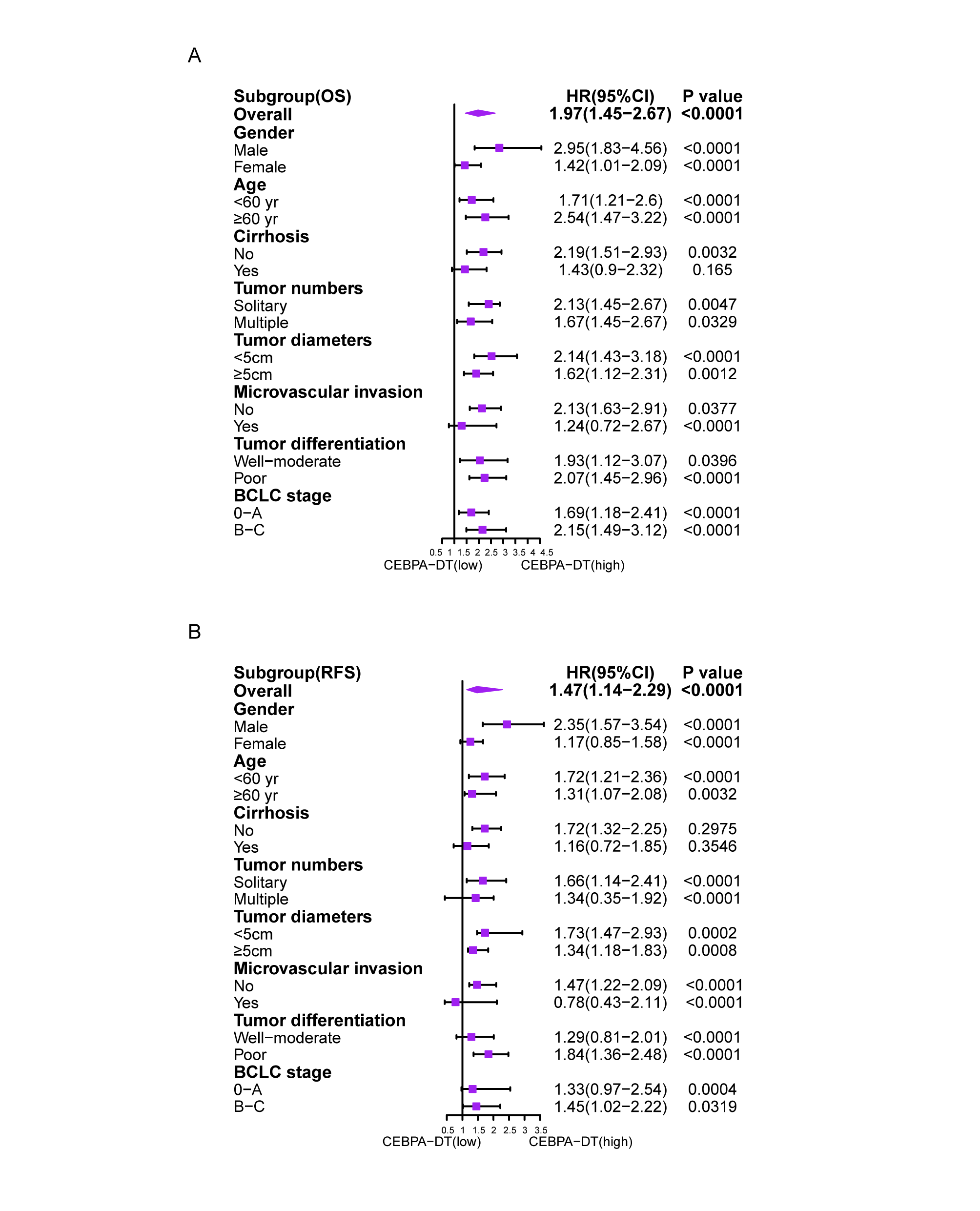

Supplement: Supplementary file 8 — Additional file 8: Fig. S7. A-B Subgroup analyses showed prognostic value of CEBPA-DT for overall survival (top) and recurrence-free survival (bottom) in HCC patients with differential features. [file 13046_2022_2544_MOESM8_ESM.tif]
